# Targeting IRAK4 disrupts inflammatory pathways and delays tumor development in chronic lymphocytic leukemia

**DOI:** 10.1038/s41375-019-0507-8

**Published:** 2019-06-13

**Authors:** Neus Giménez, Ralph Schulz, Morihiro Higashi, Marta Aymerich, Neus Villamor, Julio Delgado, Manel Juan, Mònica López-Guerra, Elias Campo, Laia Rosich, Martina Seiffert, Dolors Colomer

**Affiliations:** 1 Experimental Therapeutics in Lymphoid Malignancies Group, Institut d’Investigacions Biomèdiques August Pi i Sunyer (IDIBAPS), CIBERONC, Barcelona, Spain; 2 Anaxomics Biotech, Barcelona, Spain; 3 Division of Molecular Genetics, German Cancer Research Center (DKFZ), Heidelberg, Germany; 4 Faculty of Biosciences, Heidelberg University, Heidelberg, Germany; 5 Hematopathology Unit, Department of Pathology, Hospital Clinic, IDIBAPS, CIBERONC, Barcelona, Spain; 6 Department of Hematology, Hospital Clinic, IDIBAPS, CIBERONC, Barcelona, Spain; 7 Department of Immunology, Hospital Clinic, HSJD-HCB Immunotherapy Platform, IDIBAPS, Barcelona, Spain; 8 University Barcelona, Barcelona, Spain

**Keywords:** Targeted therapies, Preclinical research, Cell signalling, Cancer microenvironment, Toll-like receptors

## Abstract

Interleukin-1 receptor-associated kinase 4 (IRAK4) plays a critical role in Toll-like receptor (TLR) signal transduction and innate immune responses. Recruitment and subsequent activation of IRAK4 upon TLR stimulation is mediated by the myeloid differentiation primary response 88 (MYD88) adaptor protein. Around 3% of chronic lymphocytic leukemia (CLL) patients have activating mutations of *MYD88*, a driver mutation in this disease. Here, we studied the effects of TLR activation and the pharmacological inhibition of IRAK4 with ND2158, an IRAK4 competitive inhibitor, as a therapeutic approach in CLL. Our in vitro studies demonstrated that ND2158 preferentially killed CLL cells in a dose-dependent manner. We further observed a decrease in NF-κB and STAT3 signaling, cytokine secretion, proliferation and migration of primary CLL cells from *MYD88-*mutated and -unmutated cases. In the *Eµ*-TCL1 adoptive transfer mouse model of CLL, ND2158 delayed tumor progression and modulated the activity of myeloid and T cells. Our findings show the importance of TLR signaling in CLL development and suggest IRAK4 as a therapeutic target for this disease.

## Introduction

Toll-like receptors (TLRs) are the most well-known molecular pattern recognition receptors. They constitute the third essential signal for naïve B-cell activation, along with B-cell receptor (BCR) triggering and interaction with T cells [1]. In case of pathological overstimulation of the immune system, such as autoimmune diseases, B cells react to self-antigens using the BCR and TLRs [2]. Continuous TLR activation and subsequently, induction of chronic inflammation are also thought to be involved in malignant B-cell transformation, regulating both tumor cells and tumor-infiltrating innate and adaptive immune cells, including monocytes and T cells [3–5]. Thus, there is a growing interest in modulating TLR activity as a strategy to prevent uncontrolled infection and limit inflammation, and for its possible beneficial effects in chronic inflammatory diseases, autoimmune diseases, and cancer [6].

TLR signaling is mediated by the adaptor molecule myeloid differentiation primary response 88 (MYD88) which recruits and activates interleukin-1 receptor-associated kinase 4 (IRAK4), accounting for almost all biological functions of MYD88 [7]. The TLR pathway eventually activates the nuclear factor kappa B (NF-κB) and Janus kinase/signal transducer and activator of transcription 3 (JAK-STAT3) pathways to promote survival, activation, and expansion of immune cells [8, 9].

A crucial role for TLRs has recently emerged in the pathogenesis of chronic lymphocytic leukemia (CLL), a malignancy that is characterized by a progressive accumulation of mature CD19^+^CD5^+^ B cells [10]. Approximately 3% of CLL cases harbor recurrent activating *MYD88* mutations [9, 11, 12]. These mutations are the most frequent in young CLL patients and are associated with a mutated status of the variable region of the immunoglobulin heavy chain (IGHV*)* locus [11, 12]. *MYD88* mutations are predominantly clonal and considered as drivers of CLL, highlighting the relevance of TLR signaling in CLL development and evolution [13, 14]. The enhanced activity of TLRs conferred by these mutations triggers an increased production of cytokines [9], which results in the recruitment of myeloid cells and T lymphocytes, creating a favorable microenvironment [15]. Previous findings suggest a therapeutic potential for IRAK4 inhibitors for the activated B-cell-like (ABC) subtype of diffuse large B-cell lymphoma (DLBCL) presenting with *MYD88* mutations, as well as for autoimmune disorders and other malignancies that depend on TLR signaling [8, 16–18]. Herein, we aimed to evaluate the in vitro and in vivo effects of ND2158, an IRAK4 competitive inhibitor, in blocking TLR-mediated responses in CLL cells and different subsets of immune cells that constitute the CLL microenvironment. The presented results enhance the understanding of this therapeutic approach and suggest new avenues for the development of novel TLR/MYD88-mediated cancer immunotherapy strategies.

## Materials and methods

### Human samples

Peripheral blood mononuclear cells (PBMCs) from CLL patients (with ≥90% tumor B cells) and healthy donors were isolated, cryopreserved and stored within the Hematopathology collection registered at the Biobank (Hospital Clinic-IDIBAPS; R121004-094). Clinical and biological data of each patient are detailed in Supplementary Table 1. *MYD88* mutations were analyzed in previous sequencing studies [9, 19, 20].

### Mouse models

*Eµ*-TCL1 (TCL1) mice on C57BL/6 background were kindly provided by Dr. Carlo Croce (Ohio State University) [21]. *Nr4a1*^GFP^ mice and *Myd88*^−/−^ mice were a kind gift from Dr. Markus Feuerer (DKFZ, Heidelberg) [22] and Prof. Dr. Hermann-Josef Gröne (DKFZ, Heidelberg) [23], respectively. Adoptive transfer (AT) of TCL1 leukemia in C57BL/6N wild-type mice (Janvier Labs, Saint-Berthevin, France) was performed as described before [24].

### In vitro TLR stimulation

Primary CLL cells or B cells from TCL1 splenocytes were cultured with TLR ligands (0.5 µg/mL Pam3CSK4-TLR1/2, 10^8^ cells/mL HKLM-TLR2, 1 µg/mL poly(I:C)LMW-TLR3, 1 µg/mL*Salmonella typhimurium* flagellin-TLR5, 1 µg/mL FSL1-TLR2/6, 1 µg/mL Imiquimod-TLR7, 1 µg/mL ssRNA40-TLR8, and 5 µM ODN2006-TLR9) alone or with a TLR agonist mix (Pam3CSK4, HKLM, FSL1 and ODN2006) from Human TLR1-9 Agonist Kit^TM^ (InvivoGen, San Diego, CA, USA). TLR4 was not assessed due to its broad recognition pattern of microbial components. For all incubations, cells were first incubated with 10 µg/mL of polymyxin B (Sigma-Aldrich, St. Louis, MO, USA) for 20 min in order to neutralize unspecific TLR stimulation. Specific ligands for TLR10 have not yet been univocally identified. Monocytes were stimulated via TLR4 using lipopolysaccharides (LPS; Sigma-Aldrich).

### Statistical analysis

Statistical data analysis was performed using Prism 6.01 Graphpad software. Statistical analyses were performed using two-tailed nonparametric tests assuming equal variances of data. Wilcoxon matched-pairs signed-rank test was used for paired comparisons. For independent comparisons, the Mann−Whitney test was used instead. The Wilcoxon signed-rank test was used to compare sample medians to a hypothetical value. Sample size was determined based on expected variance of read-out. No samples or animals were excluded from the analyses. No randomization or blinding was used in animal studies. The statistical test used for each data set is indicated in the figure legends. Statistical significance was considered when *P* value < 0.05.

## Results

### *MYD88*-mutated CLL cases harbor an inflammatory phenotype

**Fig. 1:**
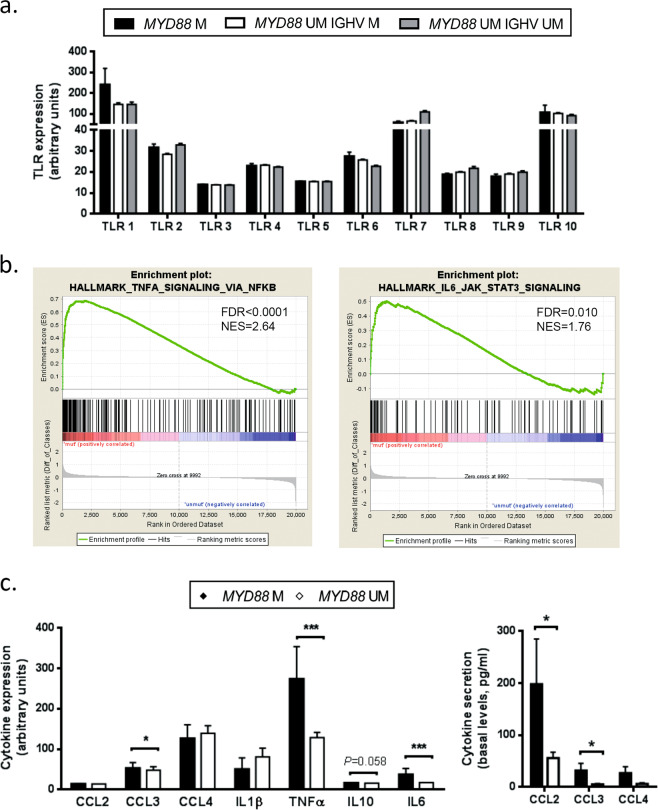
*MYD88*-mutated CLL cases harbor an inflammatory phenotype. **a** TLR gene expression profile in CLL cells comparing *MYD88*-mutated (*n* = 18 IGHV-mutated) and *MYD88*-unmutated cases (*n* = 249 IGHV-mutated and *n* = 143 IGHV-unmutated). **b** Gene set enrichment analysis comparing *MYD88*-mutated (*n* = 18) versus *MYD88*-unmutated (*n* = 249) CLL cases (all IGHV-mutated). **c** Left panel: Cytokine transcript levels of *MYD88*-mutated (*n* = 18) and *MYD88*-unmutated CLL cases (*n* = 249 IGHV-mutated CLL samples) analyzed by gene expression profile. Right panel: Cytokine secretion of CLL cells from *MYD88*-mutated (*n* = 6) and *MYD88*-unmutated (*n* = 9) cases (all IGHV-mutated) was analyzed after 48 h of culture by flow cytometry using Luminex® Bead Panel. Bars represent the mean ± SEM of all samples analyzed. Wilcoxon signed-rank test was used for statistical analysis. *P < 0.05, ***P < 0.001. Gene sets with false discovery rate (FDR) *q* value < 0.05 and a normalized enrichment score (NES) ≥ 1.5 were considered to be significantly enriched in the mutated group. M mutated, UM unmutated

As the TLR pathway has been described to have a central role in *MYD88*-mutated CLL cases [9], we first analyzed the mRNA expression of the TLR repertoire in CLL cells. By using gene expression data generated previously [19], we compared *MYD88*-mutated cases (*n* = 18, all IGHV-mutated) with *MYD88-*unmutated cases (*n* = 249 IGHV-mutated and *n* = 143 IGHV-unmutated). The TLR expression profiles in the three subgroups were very similar, with high expression levels of *TLR1*, *TLR7* and *TLR10*, and intermediate levels of *TLR2*, *TLR4*, *TLR6*, *TLR8* and *TLR9*. CLL cells expressed low levels of *TLR3* and *TLR5* (Fig. 1a).

To characterize the impact of *MYD88* mutations on the transcriptome of CLL cells, we compared gene expression profiles (GEP) of 18 *MYD88-*mutated CLL cases with 398 cases without mutation in this gene and further restricted this analysis to CLL IGHV-mutated cases (*n* = 249), as all 18 cases with *MYD88* mutations were among this group. Several gene sets related to cytokines and inflammation, such as NF-κB pathway and STAT signaling, were the most enriched in the *MYD88-*mutated subgroup compared to *MYD88*-unmutated cases (Fig. 1b, Supplementary Fig. S1, and Supplementary Table 3). Accordingly, we observed significantly higher mRNA expression levels for CCL3, TNFα, and IL6 in *MYD88*-mutated compared to -unmutated cases (Fig. 1c, left panel). In line with this, primary CLL cells from *MYD88*-mutated patients analyzed in cell culture supernatants by Luminex® Bead Panel secreted higher levels of CCL2, CCL3, and CCL4 in vitro compared with *MYD88*-unmutated CLL cases (Fig. 1c, right panel). IL1β, TNFα, IL10, and IL6 secretion levels were under the limit of detection.

### TLR stimulation modulates cytokine secretion, intracellular signaling, and CLL cell proliferation

**Fig. 2:**
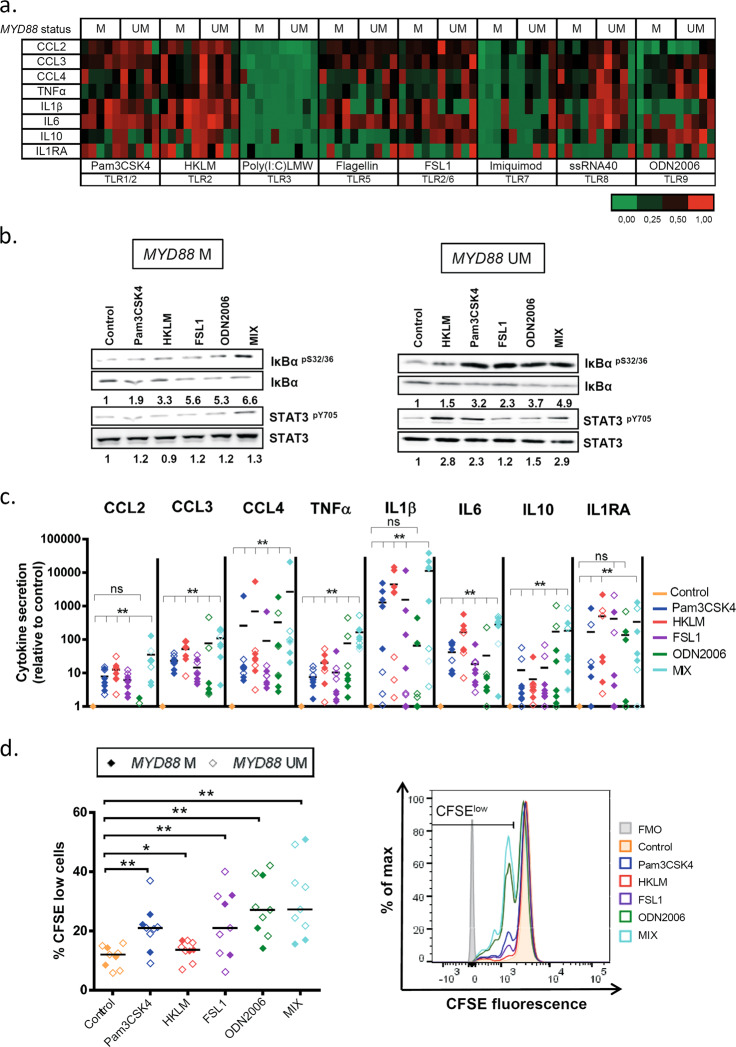
TLR stimulation increases cytokine secretion, NF-κB and STAT3 signaling and proliferation of CLL cells. CLL cells were cultured with single TLR agonists or the TLR agonist mix (Pam3CSK4, HKLM, FSL1 and ODN2006). **a** Heatmap representing cytokine secretion in CLL supernatants after 48 h of TLR stimulation of *MYD88*-mutated and *MYD88*-unmutated cases (*n* = 5 each) analyzed by flow cytometry Luminex® Bead Panel. The level of secretion of each cytokine is represented relative to each control. **b** Western blot analysis of IκBα^pS32/36^ and STAT3^pY705^ phosphorylation and total levels of IκBα and STAT3 in CLL cell extracts after 3 h of single or TLR agonist mix stimulation. Analysis of α-tubulin was used as loading control. A representative *MYD88*-mutated (#07) and *MYD88*-unmutated (#25) CLL case are shown. Ratios of phosphorylated and total protein levels were calculated and provided numbers are as fold changes relative to the untreated control sample. **c** Cytokine secretion after 48 h of TLR stimulation was assessed in cell culture supernatants of *MYD88*-unmutated (*n* = 3) and *MYD88*-mutated (*n* = 5) CLL cases by flow cytometry Luminex® Bead Panel. Data are presented as fold change relative to unstimulated control. Wilcoxon signed-rank test was used for statistical analysis. **d** Left panel: Percentage of proliferating CD19^+^ CLL cells after single or TLR agonist mix stimulation for 6 days measured by CFSE dilution (*n* = 3 *MYD88*-mutated; *n* = 6 *MYD88*-unmutated). Right panel: Flow cytometry histogram of a representative *MYD88*-unmutated CLL case (#51) shows proliferating cells (gated on viable CD19^+^ cells) after 6 days of TLR and IL15 stimulation. A decrease in CFSE signal is indicative for cells that have divided. Wilcoxon matched-paired signed-rank test was used for statistical analysis. Horizontal bars represent population means. n.s. not significant, *P* ≥ 0.05, *P < 0.05, **P < 0.01. M mutated, UM unmutated, FMO fluorescence-minus-one

We next analyzed the functionality of TLRs in CLL by stimulating primary CLL cells from *MYD88*-mutated and -unmutated cases with various TLR agonists in an attempt to distinguish the contribution of each TLR to cell signaling. CLL cells (CD19^+^CD5^+^ cell content: 95.1% ± 2.6%) were cultured with TLR agonists and levels of CCL2 (MCP1), CCL3 (MIP1α), CCL4 (MIP1β), TNFα, IL1β, IL6, IL10, IL1RA, IFNγ and IL12-p70 were analyzed in cell culture supernatants using Luminex® Bead Panel. All cytokines, besides IFNγ and IL12-p70, were detectable and upregulated by TLR stimulation (Fig. 2a). Except Poly(I:C)LMW and Imiquimod which barely induced cytokine secretion in CLL cells, all other TLR agonists triggered a strong cytokine response, with TLR1 and -2 agonists being the strongest inductors in most CLL cases. This suggests activity for TLR1, -2, -5, -6, -8 and -9, but not TLR3 and -7 in CLL cells.

We further analyzed the effect of the different TLR agonists on NF-κB and STAT3 signaling by immunoblot analysis. The highest levels of phosphorylated IκBα^pS32/36^ and STAT3^pY705^ were observed in CLL cells after stimulation with Pam3CSK4, HKLM, FSL1 and ODN2006 (TLR1, -2, -6 and -9 agonists) (Supplementary Fig. S2). As responses to TLR agonists were heterogeneous among cases, we tested the combination of these four TLR agonists (named as “mix”) to achieve a full activation of the pathway in all cases. We observed higher phosphorylation levels of IκBα and STAT3 (Fig. 2b, Supplementary Fig. S3), and higher cytokine levels in CLL cell culture supernatants (Fig. 2c) upon stimulation with this mix compared to individual TLR agonists. We further detected a stronger induction of CLL cell proliferation, analyzed by CFSE dilution, by the TLR mix (up to eightfold of unstimulated cells) compared to single TLR agonists (2- to 7-fold) (Fig. 2d). For all analyzed responses to TLR stimulation, no differences between *MYD88*-mutated and -unmutated CLL cases were observed. We also analyzed responses to TLR agonist mix in IGHV-unmutated CLL cases and obtained similar results as with IGHV-mutated CLL cells (Supplementary Fig. S4). Therefore, we used the TLR agonist mix for further analyses to achieve a complete activation of TLR signaling in CLL cells.

### The IRAK4 inhibitor ND2158 decreases viability and proliferation in CLL cells

**Fig. 3:**
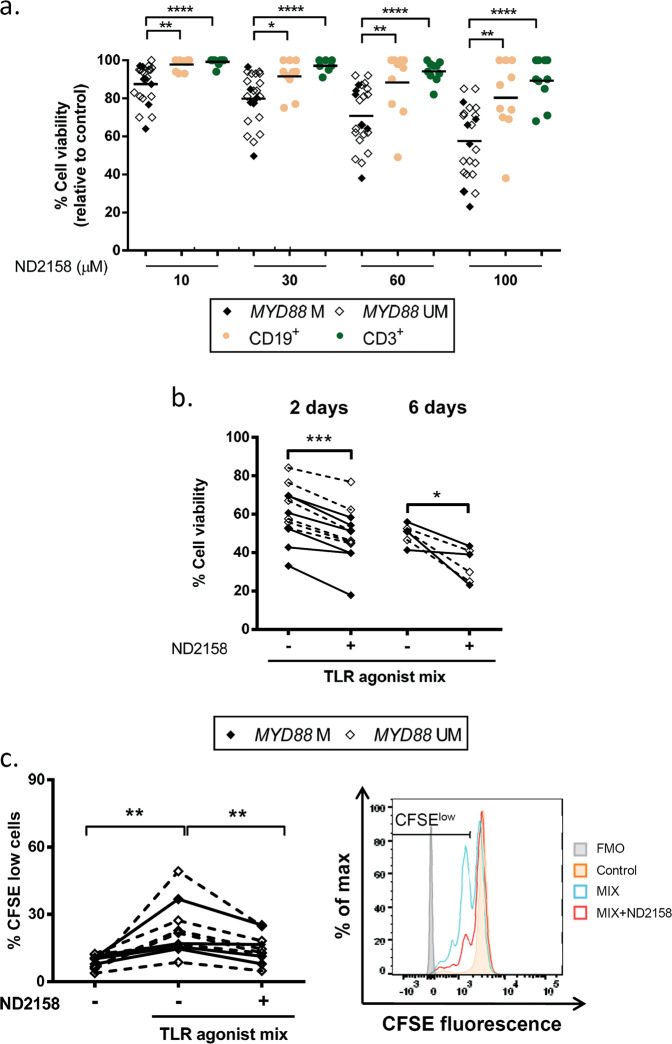
ND2158 exerts preferential cytotoxicity for CLL cells. **a** Viability was analyzed by flow cytometry after 48 h of incubation of cells in culture at ND2158 concentrations from 10 to 100 µM in *MYD88-*mutated IGHV-mutated (*n* = 6), *MYD88-*unmutated IGHV*-*mutated (*n* = 16) CLL samples, and in CD19^+^ B cells and CD3^+^ T cells from healthy donors (*n* = 10). Percentage of viable cells was measured by Annexin-V and normalized to untreated control. **b** Viability of ND2158-treated CLL cells was analyzed after TLR stimulation for 2- (*n* = 12) and 6 days (*n* = 6). **c** Left panel: Percentage of proliferating CD19^+^ CLL cells after TLR stimulation and ND2158 treatment for 6 days measured by CFSE dilution (*n* = 5 *MYD88*-mutated; *n* = 7 *MYD88*-unmutated). Right panel: Flow cytometry histogram of a representative *MYD88*-unmutated CLL case (#51) shows proliferating cells (gated on viable CD19^+^ cells) after 6 days of TLR and IL15 stimulation and ND2158 treatment. A decrease in CFSE signal is indicative for cells that have divided. Wilcoxon signed-rank test was used for statistical analysis. Horizontal bars represent population means. *P < 0.05, **P < 0.01, ***P < 0.001, ****P < 0.0001. M mutated, UM unmutated

To evaluate the potential of IRAK4 inhibition for CLL, cells from 37 CLL patients were exposed to increasing concentrations of the IRAK4 inhibitor ND2158. Treatment with 10−100 µM ND2158 significantly reduced cell viability compared to untreated samples in a dose-dependent manner (Fig. 3a). This effect was higher in CLL cells compared to B or T lymphocytes from healthy donors (Fig. 3a and Supplementary Fig. S5). There was no difference in the effect of ND2158 on CLL cells from *MYD88*-mutated and -unmutated cases, as previously reported [25]. Comparable results were obtained by analyzing intracellular ATP levels of ND2158-treated and untreated CLL cells of the same cases (Supplementary Fig. S6).

ND2158 reduced viability of CLL cells also in co-cultures with monocytes that protect CLL cells from spontaneous apoptosis in vitro and are therefore considered to model the tissue microenvironment of CLL (Supplementary Fig. S7).

We further tested the cytotoxic activity of ND2158 for CLL cells that were stimulated with TLR agonists and confirmed significantly lower levels of cell viability in treated versus untreated samples, for both *MYD88*-mutated and -unmutated cases after 2 and 6 days of incubation (Fig. 3b).

CLL cell proliferation induced by TLR agonists after 6 days was impaired by ND2158 treatment and no difference between *MYD88*-mutated and -unmutated cases was observed (Fig. 3c). Similar results were obtained using single TLR agonists to stimulate CLL cells (Supplementary Fig. S8). These results were validated by analyzing incorporation of EdU in proliferating cells and by Ki-67 stainings (Supplementary Fig. S9).

### ND2158 downregulates NF-κB and STAT3 signaling in TLR-stimulated CLL cells

**Fig. 4:**
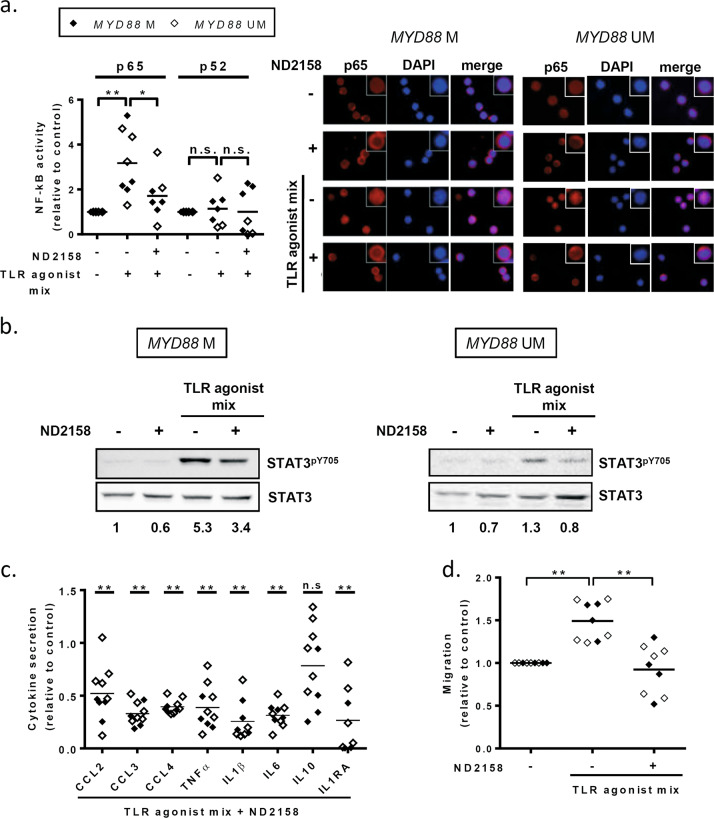
Impact of ND2158 on CLL cell signaling, cytokine release and migration. CLL cells were stimulated in vitro with TLR agonist mix for 30 min, before 10 µM ND2158 was added to the culture. **a** Left panel: Binding of p65 or p52 to NF-κB consensus sequence was analyzed using nuclear extracts from CLL cells of *MYD88*-mutated (*n* = 4) and *MYD88*-unmutated (*n* = 4) samples via a DNA-binding ELISA-based assay 3 h after treatment. Values are represented relative to untreated samples. Right panel: p65 translocation to the nucleus was analyzed by immunofluorescence microscopy in a representative *MYD88*-mutated (#7) and *MYD88*-unmutated (#19) CLL case 3 h after treatment. p65 was stained with anti-NF-κB p65 antibody (clone D14E12) for 30 min, and incubated with an Alexa546-conjugated secondary antibody (red), and DAPI (blue) was used to stain the nuclei. **b** Western blot analysis for STAT3^pY705^ of CLL cell extracts of a representative *MYD88*-mutated (#1) and *MYD88*-unmutated CLL case (#19) 3 h after treatment. Ratios of phosphorylated and total protein levels were calculated and provided numbers are fold changes relative to the untreated control sample. **c** Cytokine secretion in supernatants from CLL cells (*n* = 4 *MYD88*-mutated; *n* = 6 *MYD88*-unmutated) exposed to TLR agonist mix prior treatment with ND2158 for 48 h was analyzed by a multiplexed sandwich immunoassay based on flow cytometry using Luminex® Bead Panel. Values are presented relative to untreated control. Asterisks indicate statistical significance level relative to control. **d** Migration of TLR-stimulated CLL cells treated with ND2158 for 3 h (*n* = 4 *MYD88*-mutated; *n* = 5 *MYD88*-unmutated) towards CXCL12 was analyzed by transwell assays. Values are presented as the ratio of migrating cells and total viable cells, relative to the untreated control. Wilcoxon matched-pairs signed-rank test was used for statistical analysis. Horizontal bars represent population means. n.s. not significant; *P* ≥ 0.05, *P < 0.05, **P < 0.01. M mutated, UM unmutated

Using a DNA-binding ELISA-based assay, we analyzed p65 and p52 NF-κB activity in nuclear extracts from *MYD88*-mutated and *MYD88*-unmutated CLL samples. At baseline, we observed a higher DNA-binding activity of p65, as reported previously [9], but not of p52 in *MYD88*-mutated compared to -unmutated CLL cells (Supplementary Fig. S10). After TLR stimulation, we observed a marked increase of p65 DNA binding which was blocked by 10 µM ND2158 (Fig. 4a, left panel). No modulation of p52 binding was observed after TLR stimulation and ND2158 treatment (Fig. 4a, left panel). Accordingly, we observed by immunofluorescence microscopy that TLR agonist stimulation of both *MYD88*-mutated and -unmutated CLL cells promoted p65 translocation to the nucleus, reflecting NF-κB signaling activity (Fig. 4a, right panel). This effect was reversed by ND2158 treatment, and p65 was mainly located in the cytoplasm (Fig. 4a, right panel). Furthermore, high levels of phosphorylated STAT3^pY705^ were observed in protein extracts of CLL cells from *MYD88*-mutated and -unmutated cases after TLR stimulation, which were decreased after ND2158 treatment (Fig. 4b, Supplementary Fig. S11). In addition, we observed a significant, 5- to 7-fold decrease in the secretion of all analyzed cytokines (CCL2, CCL3, CCL4, TNFα, IL1β, IL6, and IL1RA), except for IL10, by ND2158 treatment of TLR-stimulated CLL cells compared to untreated cells (Fig. 4c). This negative impact of ND2158 on cytokine secretion was further confirmed in CLL cells that were stimulated with single TLR agonists (Supplementary Fig. S12).

We next analyzed the effect of ND2158 on VCAM-1-mediated adhesion and migration of CLL cells triggered by CXCL12, a key chemokine for CLL cell homing to lymphoid tissues [26]. We observed that cell migration considerably increased after TLR activation and that it was significantly reduced by ND2158 to a similar level as nonstimulated cells (Fig. 4d). The observed effects were comparable in cases with or without *MYD88* mutation.

We further analyzed the functional impact of ND2158 in IGHV-unmutated CLL cases and obtained similar results as with IGHV-mutated cases (Supplementary Fig. S13), validating effectivity of ND2158 in both CLL subtypes.

### ND2158 reduces viability, proliferation, and cytokine secretion of TCL1 leukemia cells in vitro

**Fig. 5:**
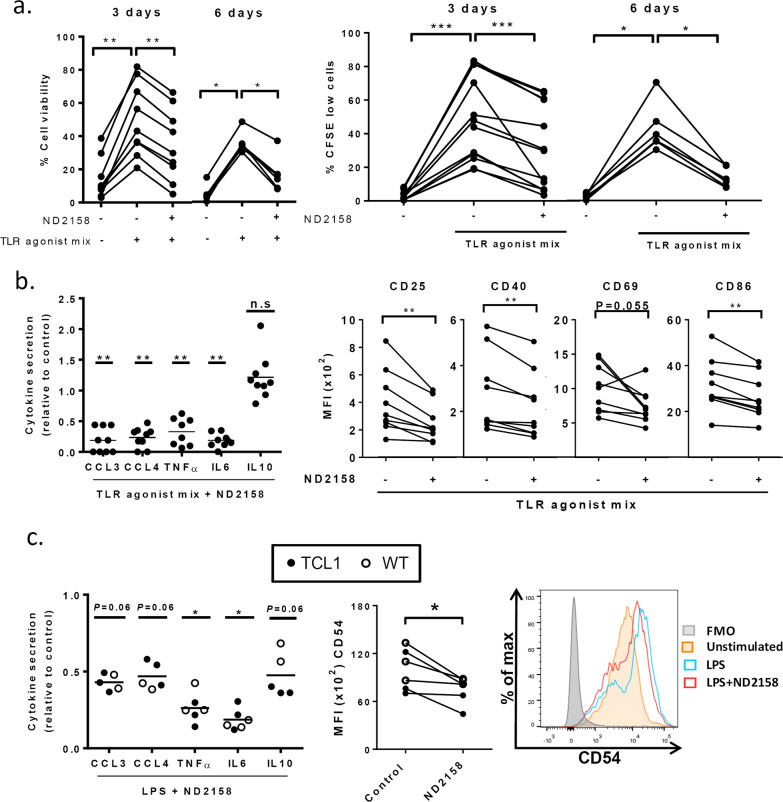
Impact of ND2158 on *Eµ*-TCL1 CLL cells and monocytes from mice. Cells were stimulated with TLR agonist mix (B cells) or LPS (monocytes) for 30 min before 10 µM ND2158 treatment. **a** Left panel: Total splenocytes of 1-year-old leukemic TCL1 mice were treated as described above, and viability of CD19^+^CD5^+^ CLL cells was analyzed after 3 days (*n* = 9) and 6 days (*n* = 6) by flow cytometry using fixable viability dye. Right panel: Percentage of proliferating CD19^+^CD5^+^ CLL cells was measured by CFSE dilution after 3 and 6 days (*n* = 6). **b** MACS-sorted B cells from spleens of 1-year-old leukemic TCL1 mice (*n* = 9) were cultured for 6 h. Left panel: Cytokine secretion data acquired as above are shown relative to untreated control. Cytokine secretion was analyzed by flow cytometry using Luminex® Bead Panel. Right panel: CD25, CD40, CD69 and CD86 expression on CD19^+^CD5^+^ CLL cells was analyzed by flow cytometry. **c** MACS-sorted monocytes from 1-year-old wild-type (WT; *n* = 3) and TCL1 mice (*n* = 3) were cultured for 6 h. Left panel: Cytokine secretion data acquired as above are shown relative to untreated control. Asterisks indicate statistical significance level relative to control. Horizontal bars represent population means. Right panel: Median fluorescence intensity (MFI) of CD54 on murine monocytes was analyzed. Wilcoxon matched-pairs signed-rank test was used for statistical analysis. n.s. not significant; *P* ≥ 0.05, *P < 0.05, **P < 0.01, ***P < 0.001

To corroborate the in vitro results obtained with primary CLL cells, we tested the effect of ND2158 on splenocytes from leukemic TCL1 mice. Upon in vitro TLR stimulation with the agonist mix, 10 µM ND2158 induced a significant reduction in cell viability in CD19^+^CD5^+^ leukemia cells after 3 and 6 days of treatment (Fig. 5a, left panel), which was also confirmed by a decrease in intracellular ATP levels in the presence of 10 and 30 µM ND2158 (Supplementary Fig. S14a). Further, ND2158 decreased the number of proliferating leukemia cells upon TLR stimulation at 3 and 6 days (Fig. 5a, right panel, and Supplementary Fig S14b, c for single TLR agonist stimulation), suggesting comparable activity of ND2158 for human and murine CLL cells.

We next analyzed cytokine secretion and activation of CD19-sorted B cells from spleens of leukemic TCL1 mice after ex vivo TLR stimulation. We observed significantly decreased secretion of CCL3, CCL4, TNFα, and IL6 in ND2158-treated versus untreated samples (Fig. 5b, left panel). In line with results from human CLL cells, ND2158 was not able to reduce the levels of TLR-induced IL10 secretion. Levels for IFNγ, IL12-p70, CCL2, and IL1β were below the detection threshold. Furthermore, ND2158 treatment reduced the expression of the B-cell activation markers CD25, CD40, CD69, and CD86 that were upregulated upon TLR stimulation (Fig. 5b, right panel).

### ND2158 affects monocyte function in vitro

Since monocytes rely for their activity on TLR-mediated signals and have been shown to support CLL cell survival [27, 28], we investigated the effect of ND2158 on these cells. Monocytes isolated from leukemic TCL1 mice or C57BL/6N wild-type mice were analyzed after ex vivo stimulation with LPS, a commonly used TLR agonist for these cells. An increase in the secretion of CCL3, CCL4, TNFα, IL6, and IL10 was observed after LPS stimulation, which was decreased after ND2158 treatment (Fig. 5c, left panel). Moreover, monocyte activation marker CD54 was upregulated after LPS stimulation and this effect was inhibited with ND2158 (Fig. 5c, right panel). These data suggest that ND2158 impairs monocyte activity and might, therefore, be able to disrupt tumor-supporting abilities of monocytes.

### ND2158 treatment delays tumor progression in the TCL1 adoptive transfer model

**Fig. 6:**
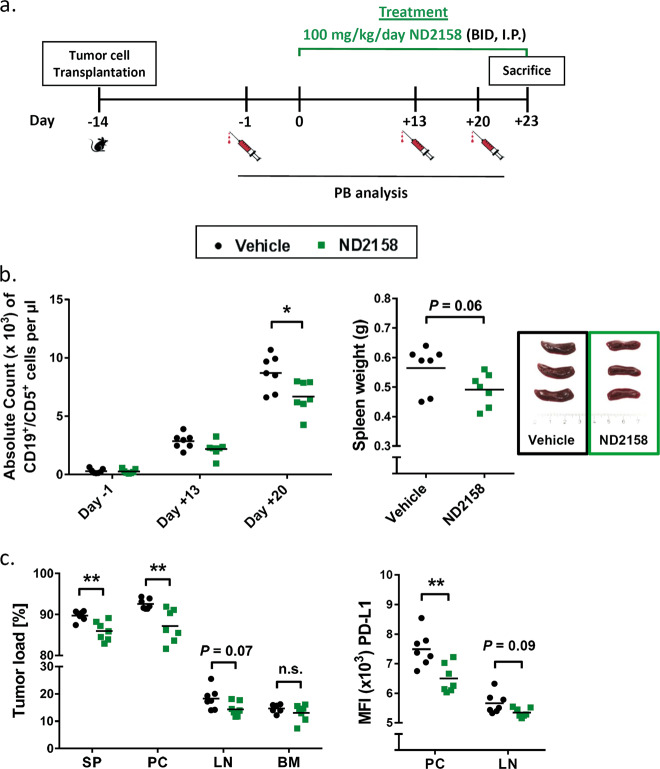
ND2158 delays CLL progression in the TCL1 adoptive transfer mouse model. **a** Treatment schedule of ND2158 in TCL1 AT model. BID twice a day, i.p. intraperitoneally. **b** Left panel: Absolute tumor cell count (CD19^+^CD5^+^) in peripheral blood (PB) over time as analyzed by flow cytometry. Right panel: Spleen weight of vehicle- (*n* = 7) and ND2158-treated (*n* = 7) mice. Representative examples of spleens are shown. **c** Left panel: Tumor load (CD19^+^CD5^+^ cells out of CD45^+^ cells) in spleen (SP), peritoneal cavity (PC), lymph nodes (LN), and bone marrow (BM) as acquired by flow cytometry. Right panel: Median fluorescence intensity (MFI) of PD-L1 in CLL cells from PC and LN. Horizontal bars represent population means. Mann−Whitney test was used for statistical analysis. *P < 0.05, **P < 0.01

In order to assess the in vivo efficacy of ND2158, we used the TCL1 AT mouse model of CLL. Splenocytes from leukemic TCL1 mice were transplanted into syngeneic immunocompetent C57BL/6N mice, assigned and treated as shown in Fig. 6a and Supplementary Fig. S15. Three weeks after the start of treatment, mice receiving ND2158 showed a significant decrease in absolute counts of CD19^+^CD5^+^ leukemia cells in PB compared to vehicle-treated controls (Fig. 6b, left panel). A similar trend was observed for absolute counts of CD19^+^CD5^+^ cells in the spleen (Supplementary Fig. S16). Mice were sacrificed after 23 days of treatment and effects in tumor-affected tissues were analyzed. ND2158 treatment reduced spleen weight (Fig. 6b, right panel) and tumor load in spleen, peritoneal cavity and lymph nodes (Fig. 6c, left panel). In addition, the expression of programmed death-ligand 1 (PD-L1), an inhibitory signal for T cells, was decreased on tumor cells in the peritoneal cavity and lymph nodes after ND2158 treatment (Fig. 6c, right panel). These data show that ND2158 is able to control leukemia progression in mice, along with a decrease in immunosuppressive features of the tumor.

### ND2158 reduces tumor-supporting monocytes in mice

**Fig. 7:**
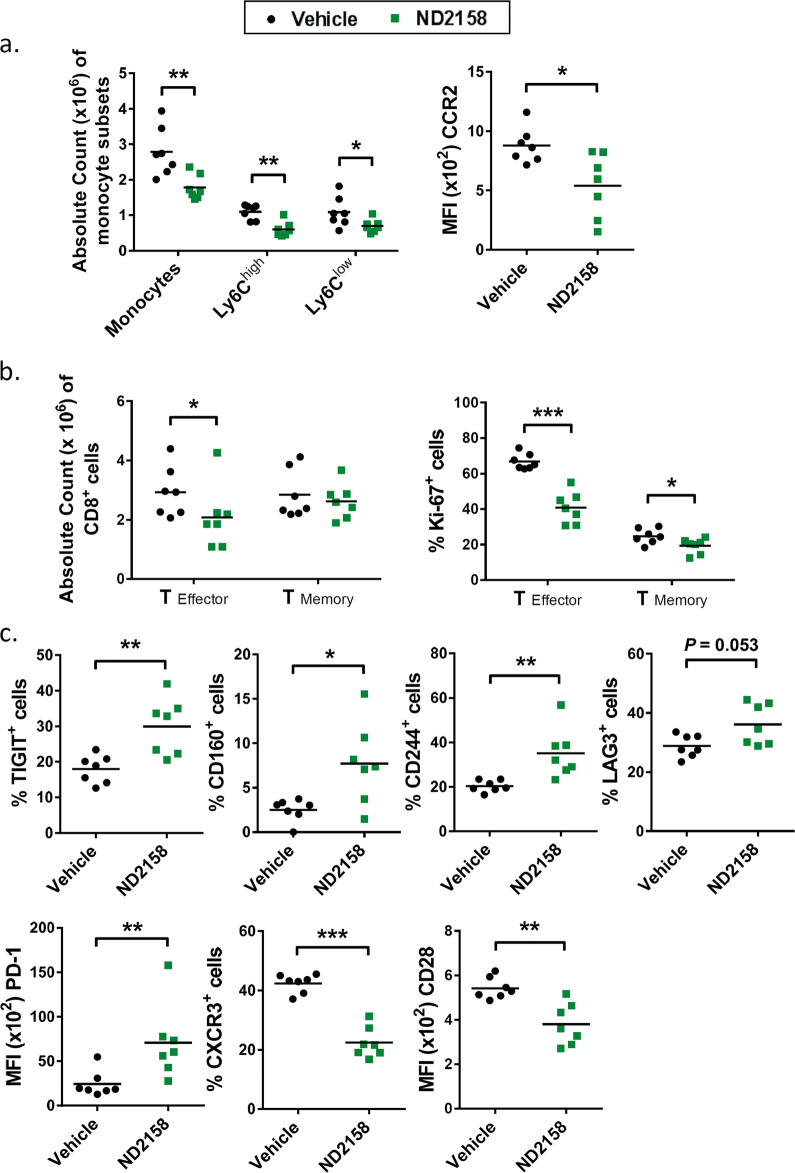
ND2158 impacts on the tumor microenvironment in the TCL1 adoptive transfer model. **a** Left panel: Absolute counts of monocytes in the spleen (SP) of vehicle- (*n* = 7) and ND2158-treated (*n* = 7) mice. Right panel: CCR2 protein expression (MFI) on Ly6C^+^ monocytes in the spleen acquired by flow cytometry. **b** Left panel: Absolute numbers of CD8^+^ effector and memory T cells in the spleen acquired by flow cytometry. Right panel: Percentage of Ki-67^+^CD8^+^ effector and memory T cells in the spleen of vehicle- and ND2158-treated mice analyzed by flow cytometry. **c** Protein expression analysis of inhibitory receptors and activation markers on CD8^+^ effector T cells by flow cytometry. Data are shown as percentage of TIGIT^+^, CD160^+^, CD244^+^, LAG3^+^, and CXCR3^+^ CD8^+^ effector T cells for bimodal populations or as MFI of costimulatory receptor CD28 as unimodal population. MFI of PD-1 was analyzed for PD-1^+^CD8^+^ effector T cells. Horizontal bars represent population means. Mann−Whitney test was used for statistical analysis. *P < 0.05, **P < 0.01, ***P < 0.001. MFI median fluorescence intensity, FMO fluorescence-minus-one

We further analyzed effects of ND2158 treatment on the immune microenvironment of mice after TCL1 AT. Thereby, we observed a significantly lower number of CD11b^+^CX3CR1^+^F4/80^+^ monocytes in ND2158-treated animals compared to the control group, and this affected both Ly6C^high^ (inflammatory) and Ly6C^low^ (patrolling) monocytes (Fig. 7a, left panel). In addition, the expression of chemokine receptor CCR2, which is important for CCL2-induced recruitment of Ly6C^high^ monocytes in this model [29], was significantly lower in these cells in ND2158-treated mice compared to vehicle-treated mice (Fig. 7a, right panel), which might indicate an impact of this drug on monocyte recruitment.

### ND2158 treatment impairs CD8^+^ T-cell proliferation and function

CD8^+^ T cells have been shown to control tumor progression in the TCL1 AT model [24]. Therefore, we analyzed the effects of ND2158 on CD8^+^ T cells in tumor-bearing mice. Interestingly, ND2158-treated mice showed significantly lower absolute numbers of antigen-experienced CD8^+^CD44^int-high^CD127^low^ effector T cells in the spleen (Fig. 7b, left panel), and a lower proportion of Ki-67^+^ cells within this population (Fig. 7b, right panel) compared to vehicle-treated animals, suggesting a lower proliferative capacity and therefore expansion of these cells by ND2158 treatment. Although no difference in absolute numbers of CD8^+^CD44^high^CD127^high^ memory T cells was observed, significantly less Ki-67^+^ cells were detected in this subset as well (Fig. 7b and Supplementary Fig. S17a). Furthermore, a significant increase in the percentage of CD8^+^ effector cells expressing the inhibitory receptors TIGIT, CD160, CD244 and LAG3 was observed in ND2158-treated mice compared to vehicle-treated animals (Fig. 7c and Supplementary Fig. S17b). We further detected a higher expression level of the immune checkpoint protein PD-1 on these cells (Fig. 7c and Supplementary Fig. S17b). Even though the percentage of PD-1^+^CD8^+^ effector T cells was similar in both treatment groups (Supplementary Fig. S17c), these cells expressed higher levels of the immune checkpoint protein PD-1 in ND2158-treated mice (Fig. 7c), which has been linked to terminal exhaustion of CD8^+^ T cells [30]. In addition, we observed a lower percentage of CD8^+^ effector T cells expressing CXCR3, a protein important for T-cell trafficking and function, and a significantly lower expression of the costimulatory receptor CD28 on these cells in ND2158-treated mice compared to controls (Fig. 7c and Supplementary Fig. S17b). These findings thus suggest a more exhausted and less functional phenotype of CD8^+^ effector T cells in ND2158-treated mice.

**Fig. 8:**
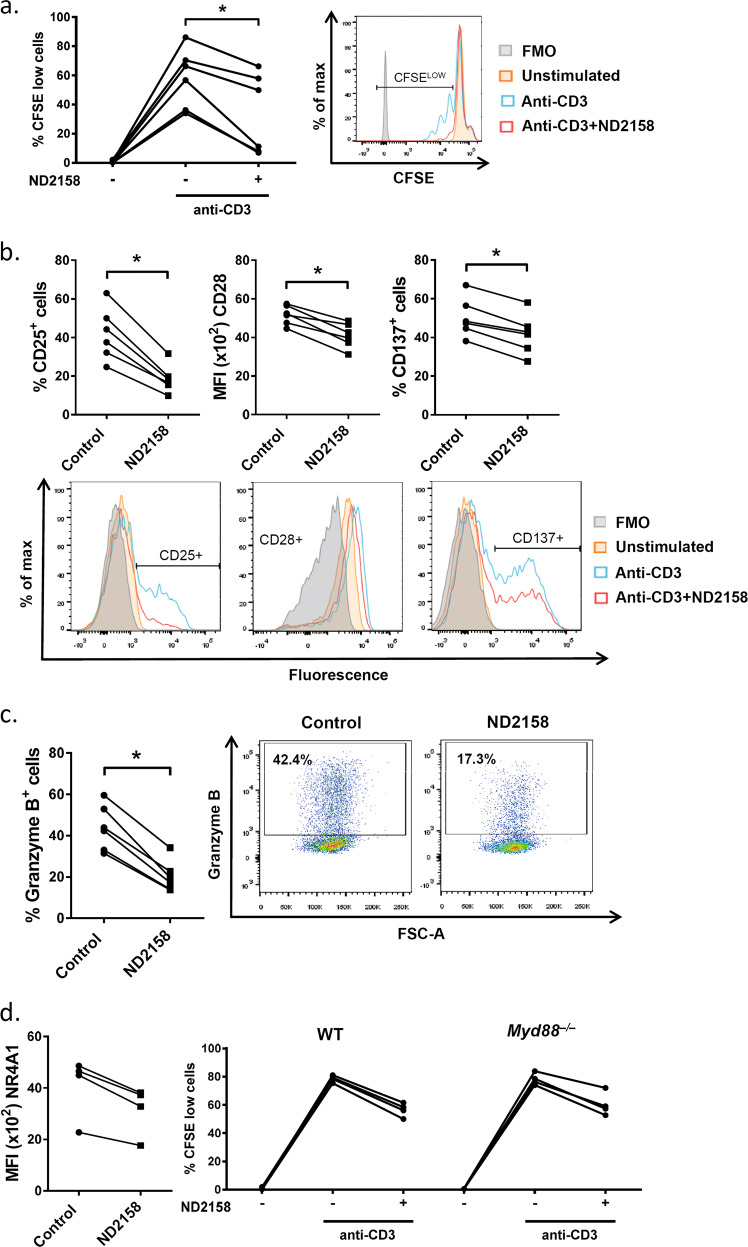
ND2158 impairs proliferation and function of CD8^+^ T cells in vitro. Cells were stimulated with an anti-CD3 antibody for 30 min followed by treatment with 10 µM ND2158. **a** Left panel: Human PBMCs from healthy donors (*n* = 6) were stained with CFSE and percentage of proliferating cells was measured in viable CD8^+^ T cells after 3 days by flow cytometry. Right panel: A representative histogram of the CFSE signal is shown. **b** Protein expression of CD25, CD28 and CD137 was analyzed after 24 h of treatment in viable CD8^+^ T cells by flow cytometry. Quantification of data is shown in the upper row; corresponding representative histograms are shown in the bottom row. Data are shown as percentage of CD25^+^ or CD137^+^ CD8^+^ T cells for bimodal populations, or as MFI of CD28 on CD8^+^ T cells as unimodal population. **c** Percentage of granzyme B^+^ viable CD8^+^ T cells was analyzed after 24 h of treatment by flow cytometry. Quantification of data is shown on the left; corresponding representative dot plots are shown on the right. Wilcoxon matched-pairs signed-rank test was used for statistical analysis. **d** Left panel: GFP expression was analyzed by flow cytometry in splenocytes from *Nr4a1*^GFP^ transgenic mice (*n* = 4) in viable CD8^+^ T cells 3 h after treatment with ND2158. Right panel: Splenocytes from wild-type (WT) C57BL/6 (*n* = 4) and *Myd88*^*–/–*^ mice (*n* = 4) were stained with CFSE and percentage of proliferating cells was analyzed in viable CD8^+^ T cells after 48 h as described above. *P < 0.05

To further follow this hypothesis, we performed in vitro experiments using anti-CD3-stimulated T cells from human PBMCs and murine splenocytes and observed a significant inhibition of proliferation of CD8^+^ T cells (Fig. 8a and Supplementary Fig. S18a), associated with lower expression of activation markers CD25, CD28 and CD137 in ND2158-treated samples compared to controls (Fig. 8b and Supplementary Fig. S18b). In addition, ND2158 significantly reduced the induction of the effector molecule granzyme B after T-cell stimulation (Fig. 8c and Supplementary Fig. S18b). We further quantified T-cell receptor (TCR) activity using transgenic *Nr4a1*^GFP^ mice, in which GFP expression is a measure of TCR signaling strength [22]. Upon anti-CD3 stimulation of *Nr4a1*^GFP^ splenocytes, we observed a considerable inhibition of GFP expression in ND2158-treated CD8^+^ T cells compared to controls (Fig. 8d, left panel and Supplementary Fig. S18c) indicating a decrease in TCR signaling.

As MYD88/IRAK4-mediated signals are not known to be of central relevance for T-cell expansion and function, we next tested whether the inhibitory effect of ND2158 on CD8^+^ T cells was dependent on this signaling complex by using anti-CD3 stimulated splenocytes from *Myd88*^*−/−*^ mice. As ND2158 inhibited proliferation, activation and granzyme B production to a similar degree in *Myd88*^*−/−*^ and wild-type CD8^+^ T cells (Fig. 8d, right panel and Supplementary Fig. S19), we suggest that the activity of ND2158 on CD8^+^ T cells might be independent of MYD88 and IRAK4.

Taken together, our results indicate impaired activation and proliferative capacity of CD8^+^ T cells in the presence of ND2158. This effect most likely contributes to the enhanced CD8^+^ T-cell exhaustion induced by ND2158 in the TCL1 AT mouse model, and thus might be a possible explanation for the only moderate effect of this drug on tumor progression in the mice.

## Discussion

CLL is a malignancy of antigen-experienced mature B lymphocytes, in which microenvironmental signals play a critical role in ontogeny and evolution [31]. Among these extracellular triggers that are known to drive CLL, auto-antigens and bacterial components which can be recognized by B cells via BCR and TLR collaboration have been described [32, 33]. Moreover, CLL patients are often associated with an increased frequency and severity of infections and autoimmune complications [34]. MYD88 is a critical adaptor protein of the TLR signaling pathway [2] and activating mutations of *MYD88* have been observed in about 3% of CLL patients [9, 12]. Our results confirm that CLL cases with mutations in *MYD88* are significantly enriched in gene expression signatures related to cytokines and inflammation, such as NF-κB and STAT3 signaling, as well as high basal cytokine secretion, which is in accordance with previous reports in CLL [12] and DLBCL [8]. CLL cells were shown to have a similar expression pattern of TLRs as normal B cells [35–38] and we did not observe differences between *MYD88*-mutated or unmutated CLL cases, indicating that the TLR signaling framework is of similar relevance in both groups. Previous studies suggested the involvement of TLR signaling in CLL cell survival [37] and its contribution to NF-κB activity and an inflammatory micromilieu in CLL [39]. In addition, response to TLR stimulation in CLL cells was shown to be dependent on biological and clinical features of patients [40, 41]. To avoid heterogeneous responses when activating TLR signaling in CLL cells, we used a mix of TLR agonists that stimulates TLR1, TLR2, TLR6, and TLR9, and showed that it reliably triggered activation of the NF-κB and JAK-STAT signaling pathways, secretion of cytokines, and enhanced CLL cell migration and proliferation in vitro in all CLL samples, highlighting the relevance of TLR signaling in CLL pathobiology.

In the last years, several IRAK4 inhibitors have been developed and tested for the treatment of cancer and other diseases related to IRAK overexpression [42]. ND2158 has been described as a highly selective and bioavailable small molecule IRAK4 inhibitor, which exhibited robust activity against the ABC subtype of DLBCL presenting with *MYD88* mutations in preclinical mouse models [16].

In CLL samples, ND2158 reduced cell viability independently of the *MYD88* mutational status, at concentrations that did not impact on normal B- and T-cell survival. ND2158 further inhibited TLR agonist-induced NF-κB and STAT3 activity, which play a cooperative role in CLL cell survival [43] and reduced viability, proliferation, adhesion−migration, and cytokine release of CLL cells which were enhanced upon TLR activation. As ND2158 also reduced the release of inflammatory factors by monocytes, which are known to support CLL progression [44], we hypothesized that its combined activity on CLL cells and myeloid bystander cells will be of benefit for the treatment of CLL.

To test the therapeutic potential of ND2158, we used the TCL1 AT mouse model of CLL that mirrors many features of human disease, including alterations in the tumor microenvironment [27, 29]. Treatment of leukemic TCL1 AT mice with ND2158 slowed down leukemia progression and led to lower tumor load in secondary lymphoid organs compared to control mice. This was accompanied with a decrease in monocyte numbers, as well as in their activation and cytokine secretion. Previous studies have shown that depletion of monocytes using clodronate-liposomes delays CLL in the TCL1 AT model, as well as in a xenotransplantation approach using the MEC-1 CLL cell line [27, 45]. Thus, the reduction and functional impairment of monocytes by ND2158 and its impact on the inflammatory milieu might reduce microenvironment-mediated support of CLL cells, and likely contributes to the observed delay in tumor progression.

Surprisingly, ND2158 further negatively impacted on CD8^+^ T-cell activity and expansion, both in vivo and in vitro. The fact that we only see a moderate effect on tumor load in our treatment study could be due to the potential negative effect of the drug on CD8^+^ T cells. In treated mice, this led to lower numbers of CD8^+^ effector T cells with reduced expression of proliferation and activation markers, and higher expression of exhaustion markers, including the inhibitory receptor PD-1, when compared to control mice. Our data further suggest that the activity of ND2158 on CD8^+^ T cells is independent of MYD88/IRAK4. An unbiased analysis of ND2158’s activity on kinases showed that besides effectively targeting IRAK4 (100% inhibition at 10 µM), ND2158 also inhibits other kinases with lower efficacy, including enzymes that are of functional relevance in T cells (e.g. > 70% inhibition of DYRK1, TXK and LCK at 10 µM) [16].

Our previous work showed that leukemia development in the TCL1 mouse model is controlled by an oligoclonally expanded CD8^+^ effector T-cell population that gradually shows features of functional exhaustion [24]. As ND2158 treatment of mice inhibits CD8^+^ effector T-cell expansion and increases their exhaustion, an inferior tumor control by these cells is expected. The results of this treatment study suggest that ND2158 enhances CD8^+^ effector T-cell exhaustion by inhibiting their proliferation. Therefore, the positive antitumor effects of ND2158 on tumor cells and monocytes seems to be counteracted by its negative impact on the T-cell compartment, which might at least partly explain the modest effects ND2158 has in controlling leukemia development in the TCL1 mouse model.

In agreement with previous studies [16, 46], we also observed a superior antitumor activity when combining the IRAK4 inhibitor ND2158 with ibrutinib or venetoclax (Supplementary Fig. S20). In addition, to overcome the negative impact of ND2158 on T cells, we propose to consider its combination with drugs that improve T-cell function and avoid their rapid exhaustion. As we observed increased expression of several inhibitory receptors on CD8^+^ T cells in ND2158-treated mice, including PD-1 and TIGIT, blocking these receptors with antibodies might be a successful strategy to overcome the loss of T-cell function and improve therapy outcome. More importantly, the development of more selective inhibitors for IRAK4 and therefore the reduced negative impact on T cells should be considered to improve therapeutic targeting of the TLR pathway in CLL and other diseases. Ideally, such drugs should not compromise the patients’ immune system, and decrease their risk of infections, which is a frequent adverse effect in treated CLL patients [16, 47].

In summary, our data suggest IRAK4 as a novel treatment target for CLL. Inhibition of IRAK4 blocks survival and proliferation of CLL cells. In addition, it impacts on the tumor-supportive inflammatory milieu, by reducing cytokine secretion in malignant and bystander cells. In light of our findings in the TCL1 mouse model, combining the IRAK4 inhibitor ND2158 with immune checkpoint therapy might result in enhanced treatment efficacies.

## Supplementary information

Online Supplementary Methods and Material
