# Supplementary material for: Targeting IRAK4 disrupts inflammatory pathways and delays tumor development in chronic lymphocytic leukemia
Source: Leukemia. 2019 Jun 13;34(1):100–14. doi: 10.1038/s41375-019-0507-8 (PMC8075947; doi:10.1038/s41375-019-0507-8)
Supplement: Supplementary file 1 — Online Supplementary Methods and Material [file 41375_2019_507_MOESM1_ESM.doc]

**Supplementary information**

**Isolation and culture of primary human cells**

Peripheral blood mononuclear cells (PBMCs) from patients diagnosed with CLL according to the World Health Organization criteria [1] and from healthy donors were used in this study. IGHV gene mutational status was assessed according to the European Research Initiative on CLL guidelines [2]. The ethical approval for this project including the informed consent of the patients was granted following the guidelines of the Hospital Clinic Ethics Committee and the Declaration of Helsinki.

Thawed cells were cultured in fresh RPMI-1640 (Gibco, Carlsbad, CA, USA) supplemented with 10% fetal bovine serum (FBS), 2 mM glutamine and 4% penicillin-streptomycin (Life Technologies) and cultured in a humidified atmosphere at 37ºC containing 5% carbon dioxide. CD14^+^ monocytes from healthy donor PBMCs were isolated via magnetic activated cell sorting (MACS) using human CD14 MicroBeads (MiltenyiBiotec Inc., Auburn, CA, USA), according to manufacturer’s protocol.

**Gene expression analysis**

Total RNA was isolated from these samples using TRIzol reagent (Life Technologies, Eugene, OR, USA) according to manufacturer's instructions. RNA integrity was examined with the Bioanalyzer 2100 (Agilent Technologies, Santa Clara, CA, USA) and only high quality samples were hybridized to AffymetrixGeneChip HT HG-U219 perfect-match-only array plate, following Affymetrix standard protocols. The Expression Console software (Affymetrix, Santa Clara, CA, USA) was used to get the summarized expression values by the Robust Multi-array Analysis. Expression array data have been submitted to the European Genome-Phenome Archive under accession number EGAS00001000772.

The gene expression profile (GEP) of *MYD88-*mutated cases versus those without mutations was compared using the gene set enrichment analysis (GSEA) package version 3.0. A respective analysis was performed including only IGHV-mutated patients. An enrichment of gene set signatures was evaluated using the Hallmark gene sets collection version 6.1 with a two class analysis, 1000 permutations of gene sets and weighted metrics. Gene sets with false discovery rate (FDR) q-value <0.05 were considered to be significantly enriched in the mutated group.

**Isolation and culture of cell suspensions from murine spleen**

Spleens from age- and sex-matched leukemic TCL1, C57BL/6 wild-type (WT) control mice, *Nr4a1*^GFP^ transgenic mice, *Myd88^–/–^* mice and vehicle- or ND2158-treated TCL1 AT mice were isolated and homogenized with a gentle MACS Dissociator (MiltenyiBiotecInc.). Single cell suspensions were obtained after erythrocyte lysis by incubation with Red blood cell lysis buffer (BioLegend, San Diego, CA, USA) [3] and filtration through 70 µm nylon sieves (BD Falcon™, Franklin Lakes, NJ, USA). For *in vitro* stimulation experiments, purified B cells, monocytes or total splenocytes were cultured in RPMI-1640 supplemented with 10% FBS and 1% penicillin/streptomycin and cultured in a humidified atmosphere at 37ºC containing 5% carbon dioxide. Purified B cells and monocytes were isolated from total splenocytes via MACS using mouse CD19 MicroBeads (MiltenyiBiotec Inc.), or the EasySep™ Mouse Monocyte Isolation Kit (StemCell Technologies, Vancouver, Canada), respectively, according to manufacturer’s protocols. Human PBMCs and murine splenocytes from C57BL/6, *Myd88^–/–^* and *Nr4a1*^GFP^ transgenic mice for CD8^+^ T cell experiments were cultured in RPMI-1640 supplemented with 10% fetal calf serum, 1% L-Glutamine, 1% non-essential aminoacids, 10 mM HEPES, 1 mM sodium pyruvate, 1x (55 µM) 2-mercaptoethanol and 1% penicillin/streptomycin.

**Cytokine measurements**

Milliplex MAP Human Cytokine Magnetic Bead Panel (Merck-Millipore, Billerica, MA, USA), a multiplexed sandwich immunoassay based on flow-cytometry Luminex® technology, was used to measure CCL2/MCP1, CCL3/MIP1α, CCL4/MIP1β, TNFα, IL1β, IL6, IL10, IL1RA, IFNγ and IL12-p70 in supernatants from PBMCs of CLL primary cases seeded at 2x10^6^ cells/mL for 48 h. B cells and monocytes from spleens of leukemic TCL1 AT mice were cultured at 2x10^6^ cells/mL for 48 h and concentrations of CCL2/MCP1, CCL3/MIP1α, CCL4/MIP1β, TNFα, IL1β, IL6, IL10, IFNγ and IL12-p70 in the culture supernatants were determined by using the fluorescence-activated cell sorter analysis (Luminex® 100 System) Milliplex MAP Mouse Cytokine Magnetic Bead Panel (Merck-Millipore). Data was analyzed with the Luminex® Xponent software.

**Western blot analysis**

Whole protein extraction and Western blot analysis were carried out as previously described [4]. Membranes were probed with antibodies against IκBα**^pS32/36^**, IκBα, STAT3**^pY705^** and STAT3 (Cell Signaling Technology, Danvers, MA, USA). Antibody binding was detected using secondary peroxidase-labeled anti-mouse and anti-rabbit (Sigma-Aldrich) antibodies and enhanced chemiluminescent substrate (ECL; ThermoScientific, Rockford, IL, USA). Chemiluminescence was detected using a mini-LAS4000 Fujifilm device (Fujifilm, Minato, TY, Japan). As two different membranes for the detection of phosphorylated proteins and total proteins were used, densitometry studies were performed by normalizing values to α-tubulin (Sigma-Aldrich) bands in each membrane, and then, calculating the ratio between normalized phosphorylated and total protein values using the Image Gauge software (Fujifilm). Subsequently, these values were normalized to results of untreated control samples. Results of α-tubulin analysis have been removed from the main figures, but the original blots including α-tubulin are shown in the supplementary figures.

***In vitro* cell proliferation assays**

Cells were labeled with 0.5 μM carboxyfluoresceinsuccinimidyl ester (CFSE; Life Technologies) and seeded in 96-well plates at a density of 10^5^ cells/200 μl. B cells were cultured in RPMI-1640 medium which was supplemented with 15 ng/ml recombinant human or murine IL15 (R&D systems, Minneapolis, MN, USA) as described before [5, 6]. When specified, cells were incubated with TLR agonists and/or ND2158. For T cell proliferation assay, PBMCs were stimulated for 30 min with 1 µg/ml anti-CD3 antibody before ND2158 addition.

The percentage of dividing cells was determined as the percentage of live human B (CD19^+^) or T cells (CD3^+^CD8^+^) or as the percentage of live tumor cells (CD45^+^CD19^+^CD5^+^) showing a decrease in CFSE signal compared to non-stimulated cells.

For 5-ethynyl-2´-deoxyuridine (EdU) incorporation, cells were cultured in medium as indicated above supplemented with 10 µM EdU (Life Technologies, Carlsbad, USA) for 6 days. For EdU detection, cells were washed and stained for surface molecules in FACS buffer as described in the Immunostainings section. Cells were fixed with 4% PFA in PBS for 15 min at room temperature, washed twice with 1% BSA in PBS and permeabilized using 0.1% Triton X-100 in PBS for 30 min at room temperature. After washing with 1% BSA in PBS, cells were incubated with 100 µl Click-iT™ reaction cocktail (9% of 10X Click-iT™ Cell Reaction Buffer, 2% of 100 mM CuSO_4_, 0.5% Fluorescent dye Alexa Fluor® 488 azide, 1% of 10X Reaction Buffer Additive in H_2_O; all from Life Technologies, Carlsbad, USA) for 30 min at room temperature in the dark. Cells were washed twice and resuspended in 1% BSA in PBS before acquisition. The percentage of proliferating cells was determined as percentage of EdU^+^ live tumor cells (CD45^+^CD19^+^CD5^+^).

Ki-67 staining of human CLL cells was performed as described in the Immunostainings section. The percentage of proliferating cells was determined as percentage of Ki-67^+^ live tumor cells (CD45^+^CD19^+^CD5^+^).

For all the assays, Fluorescence-minus-one (FMO) was used as a negative control. Data analysis was performed using FlowJo v 10.0.7 software (FlowJo, Ashland, OR, USA).

**Analysis of cytotoxicity**

ND2158 was provided by Nimbus Therapeutics (Cambridge, MA, USA). Human PBMCs from healthy donors or from 37 CLL patients with ≥90% tumor B cells were incubated for 48 h with ND2158 at doses ranging from 10 to 100 µM. Cell death was quantified by flow cytometry after staining with Annexin-V-FITC and propidium iodide (PI) (eBiosciences, San Diego, CA, USA). B and T lymphocytes from healthy donor samples were identified by staining with anti-CD3 and -CD19 antibodies (Becton Dickinson) and cytotoxicity determined by Annexin-V-PB (Life-Technologies). Percentage of viable cells was defined as Annexin-V^-^ cells. Viability of TCL1 splenocytes was determined by Fixable Viability Dye eFluor 780 (Via780; eBiosciences), ~~which was added to cell suspensions at 1:1000~~, as the percentage of CD45^+^CD19^+^CD5^+^Via780^-^ cells. Fixable Viability Dye eFluor 780 (eBiosciences) was added 1:1000 diluted for live/dead cell discrimination.

**CellTiter-Glo luminescent cell viability assay**

Viable cells in culture were determined using the CellTiter-Glo® Luminescent Cell Viability Assay (Promega, Madison, WI, USA), based on quantitation of intracellular ATP levels, following manufacturer’s instructions. Primary CLL cells were incubated with 10 µM or 30 µM ND2158 for 48 h assay performance. Data is presented relative to untreated control.

**NF-κB DNA-binding assay**

Nuclear extracts were generated from primary CLL cells and assayed for NF-κB p65 and p52 activity using the Nuclear Extract Kit and the TransAM NF-κB Chemiluminescence kit (Active Motif, Carlsbad, CA, USA), respectively. Two micrograms of nuclear extracts were incubated for 3 hours according to the manufacturer’s protocol in 96-well plates coated with an oligonucleotide containing the NF-κB consensus DNA-binding site. DNA binding of NF-κB subunits was detected by incubating with an antibody against p65 or p52 followed by a horseradish peroxidase-conjugated secondary antibody (ELISA-based method). The acquisition and quantification of the signal were done on a LAS4000 device (Fujifilm).

**Immunofluorescent microscopy**

For immunofluorescent staining, CLL cells were washed in PBS, fixed in 4% paraformaldehyde and attached to poly-L-lysine-coated cover glass slides overnight at 4ºC. After washing the slides with PBS, bound cells were permeabilized with 0.1% saponine in PBS, washed twice in PBS, incubated with anti NF-κB p65 antibody (clone D14E12; Cell Signaling Technology) for 30 min, washed twice in PBS, and incubated with an Alexa546-conjugated secondary antibody (Invitrogen, Carlsbad, CA, USA). The cells were washed three times in PBS and mounted in anti-fading mounting reagent including DAPI (Sigma-Aldrich). Stained cells were imaged using a fluorescent microscope (Eclipse 50i; Nikon, Corp., Tokyo, Japan) equipped with a CCD camera (CoolCube1, MetaSystems Hard & Software GmbH, Altlussheim, Germany) and a precentered fiber illuminator as light source. Oil immersion objective lens of 100x was used for imaging of cells.

**Chemotaxis assay**

CLL cells were washed twice and maintained in serum-starved in FBS-free RPMI during the whole experiment. When indicated, TLR agonist mix was added 30 min before ND2158 treatment. Three hours after treatment cells were diluted to 5×10^6^ cells/mL with 0.5% bovine serum albumin (BSA; Sigma-Aldrich) in PBS. One hundred microliters of the cell suspension (5×10^5^ cells) were added to the top chamber of a Transwell culture polycarbonate insert with 6.5 mm diameter and 5 μm pore size (Corning, Corning, NY, USA). Transwell inserts had been previously coated with VCAM-1 (Peprotech, Rocky Hill, NJ, USA) overnight, washed twice with PBS, and transferred to 24-well culture plates containing 600 μL of RPMI with 0.5% BSA with or without 200 ng/mL of human recombinant CXCL12 (Peprotech) per well. After 3 h of incubation, 100 μL from each lower chamber of the transwell plate were collected in triplicate and viable cells counted on a cytometer for 12 s under a constant flow rate of 500 µL/min. Values are presented as the ratio of migrating cells and total viable cells, relative to the untreated control. The untreated condition shows CXCL12-induced migration of unstimulated CLL cells which was also reduced by ND2158 treatment in some cases.

**TCL1 Adoptive Transfer (AT) mouse model**

Eight-week-old female C57BL/6N wild-type mice (Janvier Labs, Saint-Berthevin, France) were injected intravenously with 1×10^7^ splenocytes from leukemic *Eµ*-TCL1 mice. Splenocytes used for the adoptive transfer had a purity of 96% CD19^+^CD5^+^ cells. After 13 days, mice were assigned to treatment arms based on tumor load in peripheral blood and injected intraperitoneally twice daily (BID) with 50 mg/kg ND2158 in 1% β-cyclodextrin (n=7) or 1% β-cyclodextrin (vehicle; n=7). Tumor progression was monitored by blood withdrawal and quantification of CD19^+^CD5^+^ cells as described below, until mice were euthanized after 23 days of treatment. Spleens were weighed, and single cell suspensions were obtained from peripheral blood, bone marrow, inguinal lymph node, spleen and peritoneal cavity. Bone marrow cells were flushed out from one femur with 5 mL of PBS with 2% FBS followed by filtration through 70 µm nylon sieves. Single cell suspensions from one inguinal lymph node were obtained by manual dissociation with a 3 mL syringe plunger in 5 mL of PBS with 2% FBS over a 70 µm nylon sieve. Single cell suspensions from peritoneal cavity were obtained by flushing peritoneal cavity with 3 mL of PBS with 2% FBS. Tumor load in the affected organs was determined via flow cytometry as absolute number or percentage of CD5^+^CD19^+^ cells. The total viable spleen cell count and the percentage of splenic monocyte or CD8^+^ T cell populations out of total viable CD45^+^ cells, analyzed by flow cytometry, were used for the calculation of absolute cell counts: (Total viable spleen cell count/100) x Percentage (%) of cell population of interest. Cell counting was performed on an automated Vi-CELL XR hemocytometer (Beckman Coulter, High Wycombe, UK). Whole blood from treated mice was stained by incubation for 30 min at 4ºC with a 1:200 diluted antibody cocktail. Erythrocyte lysis and cell fixation were done using the 1-step Fix/Lyse Solution (eBioscience). 123count eBeads™ Counting Beads (eBioscience) were added before cell acquisition to determine the absolute cell counts per µL. Absolute cell numbers in blood were calculated according to the formula: absolute count (cells/μL) = (cell count x bead volume x bead concentration)/(bead count x cell volume). All *in vivo* experiments were performed at the German Cancer Research Center (DKFZ) according to local animal experimental ethics committee guidelines and after approval by the Regierungspräsidium Karlsruhe.

**Immunostainings for flow cytometry**

At experimental endpoints, single cell suspensions of all analyzed tissues were washed with FACS buffer (PBS containing 2% FBS) and incubated with the respective surface marker antibody cocktails for 30 min at 4°C in the dark. BD Horizon™ Fixable Viability Stain 700 (BD Biosciences, Franklin Lakes, NJ, USA), Fixable Viability dye eFluor 506 (eBioscience) or Fixable Viability Dye eFluor 780 (eBioscience) was added at 1:1000 dilution for live/dead cell discrimination. Cells were then washed twice with FACS buffer, fixed in 100 µl IC fixation buffer (eBioscience) for 30 min at room temperature and washed with FACS buffer again. Cells were kept at 4°C in the dark until acquisition. For intracellular staining, fixed cells were permeabilized in 200 µl of 1x Permeabilization buffer (eBioscience) for 5 min at room temperature and then incubated with intracellular marker antibody cocktail in 1x Permeabilization buffer for 30 min at room temperature in the dark. For nuclear Ki-67 stainings, cells were fixed and permeabilized in 200 µl Foxp3/Transcription Factor Fixation/Permeabilization buffer (eBioscience) for 30 min at room temperature followed by incubation with an anti-mouse or anti-human Ki-67 antibody in 1x Permeabilization buffer for 30 min at room temperature in the dark. Cells were washed twice with FACS buffer and cells were kept at 4°C in the dark until acquisition.

***In vitro* T cell stimulation**

Human PBMCs and murine splenocytes were incubated with 1 µg/ml anti-human CD3e (Clone UCHT1; Biolegend) and 1 µg/ml anti-mouse CD3e (Clone 145-2C11; eBioscience) antibody, respectively. For detection of granzyme B and cytokines, cells were restimulated with 0.5x PMA/ionomycin stimulation cocktail (eBioscience) in the presence of 1x protein transport inhibitor cocktail (eBioscience) for 4 h before harvesting the cells. T cell activation was assessed after 24 h of stimulation.

**Flow cytometry sample acquisition and data analysis**

Samples were acquired either on a FACS-Canto II flow cytometer (BD Biosciences) using FACS-DIVA 6.1.1 software, a BD LSR Fortessa flow cytometer using the BD FACSDiva software version 8.0.2 or an Attune focusing acoustic cytometer (Life Technologies). Data analyses were performed using FlowJo10.0.7 software. Bimodal populations were quantified as percentage of protein-expressing cells, whereas for unimodal populations mean fluorescence intensities (MFI) were analyzed. MFI were recorded and normalized by subtracting the MFI of the respective FMO control.

Gating strategies for analyzed immune subsets in the spleen were as follows: cell debris and doublets were excluded from the analysis and only live cells were selected for further analysis. Murine splenic monocytes were identified as CD45^+^Lineage^-^Ly6G^-^CD11b^+^CX3CR1^+^F4/80^+^ cells. Lineage markers included CD19, CD3, NK1.1 and TER-119. Within this population, inflammatory monocytes were defined as Ly6C^high^ and patrolling monocytes as Ly6C^low^ cells. Splenic cytotoxic T cells were identified as CD45^+^CD3^+^CD8^+^ cells. Identification of CD8^+^ T cells subsets was based on the expression of CD127 and CD44, with CD8^+^ effector T cells identified as CD127^low^/CD44^int-high^ cells and CD8^+^ memory T cells as CD127^high^CD44^high^ cells.

**References**

1. Swerdlow SH, Campo E, Pileri SA, Harris NL, Stein H, Siebert R, et al. The 2016 revision of the World Health Organization classification of lymphoid neoplasms. Blood. 2016;127:2375-90.

2. Ghia P, Stamatopoulos K, Belessi C, Moreno C, Stilgenbauer S, Stevenson F, et al. ERIC recommendations on IGHV gene mutational status analysis in chronic lymphocytic leukemia. Leukemia. 2007;21:1-3.

3. Hoyer KK, French SW, Turner DE, Nguyen MT, Renard M, Malone CS, et al. Dysregulated TCL1 promotes multiple classes of mature B cell lymphoma. Proc Natl Acad Sci U S A. 2002;99:14392-7.

4. Giménez N, Martínez-Trillos A, Montraveta A, Lopez-Guerra M, Rosich L, Nadeu F, et al. Mutations in RAS-BRAF-MAPK-ERK pathway define a specific subgroup of patients with adverse clinical features and provide new therapeutic options in chronic lymphocytic leukemia. Haematologica. 2018; e-pub ahead of print 27 September 2018; doi: 10.3324/haematol.2018.196931.

5. Mongini PK, Gupta R, Boyle E, Nieto J, Lee H, Stein J, et al. TLR-9 and IL-15 Synergy Promotes the In Vitro Clonal Expansion of Chronic Lymphocytic Leukemia B Cells. J Immunol. 2015;195:901-23.

6. Gupta R, Yan XJ, Barrientos J, Kolitz JE, Allen SL, Rai K, et al. Mechanistic Insights into CpG DNA and IL-15 Synergy in Promoting B Cell Chronic Lymphocytic Leukemia Clonal Expansion. J Immunol. 2018;201:1570-85.

**SUPPLEMENTARY TABLES**

| **# Patient** | **Gender/**  **Age at diagnosis** | **^a^%Tumor cells** | **^b^Binet/**  **Rai stage** | ***^c^*IGHV** | **Previous treatment** | **^d^Cytogenetic alterations** | ***MYD88* Mutational status** | **^e^Other Recurrent mutations** | **ICGC** |
| --- | --- | --- | --- | --- | --- | --- | --- | --- | --- |
| **CLL 01** | M/45 | 98 | C/IV | M | No | del(13q) | L265P | *KLHL6* | 3 |
| **CLL 02** | M/49 | 96 | C/IV | M | No | del(13q) | L265P | *CHD2* | 181 |
| **CLL 03** | F/43 | 96 | A/I | UM | Flu | - | M | *-* | 553 |
| **CLL 04** | M/56 | 95 | B/III | M | No | del(13q), TRISOMY12 | M232T | *SF3B1* | 564 |
| **CLL 05** | M/37 | 93 | B/II | M | No | - | L265P | *-* | 629 |
| **CLL 06** | M/43 | 90 | B/II | M | Cl | del(13q) | L265P | *-* | 633 |
| **CLL 07** | M/57 | 94 | B/II | M | Cl | - | V209F | *-* | 1534 |
| **CLL 08** | F/53 | 94 | B/II | UM | Flu,R-FCM,B-R | del(13q),del(11q) | UM | *IKZF3,POT1,*  *SF3B1* | 13 |
| **CLL 09** | M/70 | 95 | B/II | UM | No | del(13q) | UM | *ATM, SF3B1* | 10 |
| **CLL 10** | M/59 | 95 | A/0 | UM | No | - | UM | *ATM,NFKBI, ZNF292,ZMYM3* | 16 |
| **CLL 11** | M/69 | 96 | BII | UM | R-CHOP,FC,Cl | TRISOMY12 | UM | *-* | 24 |
| **CLL 12** | M/53 | 97 | C/IV | UM | R-FCM | del(13q) | UM | *TLR2,POT1, ZNF292* | 44 |
| **CLL 13** | M/52 | 97 | B/II | UM | Yes | del(13q) | UM | *-* | 63 |
| **CLL 14** | M/64 | 93 | B/II | M | No | TRISOMY12,  t(14;18) | UM | *-* | 64 |
| **CLL 15** | M/52 | 94 | B/I | UM | No | del(17p) | UM | *-* | 75 |
| **CLL 16** | M/47 | 86 | C/IV | UNKN | multiple | del(13q),  der(11)t(11;13) | UM | *-* | 79 |
| **CLL 17** | M/58 | 98 | B/II | UM | multiple | del(11q) | UM | *-* | 101 |
| **CLL 18** | M/62 | 98 | C/IV | M | B | del(13q) | UM | *-* | 115 |
| **CLL 19** | M/66 | 95 | C/IV | M | No | del(13q) | UM | *-* | 159 |
| **CLL 20** | M/65 | 94 | A/I | UM | No | del(13q) | UM | *-* | 186 |
| **CLL 21** | M/58 | 93 | A/I | M | No | - | UM | *PTPN11* | 191 |
| **CLL 22** | M/56 | 93 | A/0 | M | No | - | UM | *-* | 273 |
| **CLL 23** | M/61 | 96 | C/III | UM | No | del(11q) | UM | *BIRC3(DEL), MED12* | 278 |
| **CLL 24** | M/78 | 94 | A/II | UM | No | del(13q) | UM | *-* | 316 |
| **CLL 25** | F/43 | 95 | A/0 | M | No | del(13q) | UM | *-* | 344 |
| **CLL 26** | M/44 | 97 | B/II | UM | No | del(13q) | UM | *XPO1* | 350 |
| **CLL 27** | F/48 | 91 | A/0 | M | No | del(13q) | UM | *-* | 361 |
| **CLL 28** | F/54 | 91 | CIII | UM | No | del(11q), del(13q) | UM | *-* | 384 |
| **CLL 29** | M/62 | 97 | B/II | UM | No | del(11q), del(13q) | UM | *ATM* | 442 |
| **CLL 30** | F/41 | 99 | C/IV | UM | Cl,Flu | del(13q) | UM | *-* | 540 |
| **CLL 31** | M/69 | 98 | A/0 | M | No | - | UM | *-* | 561 |
| **CLL 32** | F/69 | 98 | A/0 | M | C | - | UM | *CHD2* | 569 |
| **CLL 33** | F/52 | 92 | B/II | M | No | TRISOMY12 | UM | *-* | 642 |
| **CLL 34** | M/54 | 96 | B/II | M | FCM, R-FCM | del(13q) | UM | *-* | 680 |
| **CLL 35** | M/59 | 95 | B/II | M | No | del(13q) | UM | *NFKBIA* | 684 |
| **CLL 36** | M/53 | 88 | A0 | M | No | - | UM | *SF3B1* | 758 |
| **CLL 37** | M/66 | 98 | B/III | UM | No | del(11q) | UM | *ATM, BIRC3(DEL)* | 761 |
| **CLL 38** | M/57 | 98 | A/0 | M | No | del(13q) | UM | *-* | 815 |
| **CLL 39** | F/56 | 94 | A/0 | M | No | del(13q) | UM | *-* | 1103 |
| **CLL 40** | F/54 | 92 | B/II | M | No | T(14;18) | UM | *-* | 1291 |
| **CLL 41** | F/63 | 97 | C/IV | M | No | del(13q) | UM | *NFKB2* | 1323 |
| **CLL 42** | M/83 | 60 | A0 | M | No | del(11q); del(13q), del(17p) | UM | *-* | 1339 |
| **CLL 43** | M/78 | 96 | A/0 | M | No | del(13q) | UM | *-* | 1481 |
| **CLL 44** | F/64 | 91 | A/0 | UM | Cl | del(11q) | UM | *-* | - |
| **CLL 45** | F/47 | 86 | A/I | UM | FCM | - | UM | *-* | 77 |
| **CLL 46** | M/56 | 98 | C/IV | UM | No | - | UM | *NOTCH1* | 11 |
| **CLL 47** | M/58 | 96 | B/II | UM | FCM; R-FCM | del(13q) | UM | *BRAF* | 27 |
| **CLL 48** | M/55 | 79 | A/0 | UM | No | t(5;14)(q34;q11.2) | UM | *NOTCH1* | 15 |
| **CLL 49** | M/60 | 79 | B/II | UM | No | del(11q) | UM | *ATM, BIRC3,SF3B1* | 306 |
| **CLL 50** | M/62 | 95 | A/0 | UM | No | TRISOMY12 | UM | *ATM, BIRC3,NOTCH* | 723 |
| **CLL 51** | M/55 | 91 | A/0 | M | No | N | UM | *-* | - |

**Table S1. Clinical and biological characteristics of CLL patients.**

Abbreviations: M, male; F, female; ND, not determined; M, mutated; UM, unmutated; del, deletion; T, treated; U, untreated; FCM, Fludarabine, Cyclophosphamide, Mitoxantrone; CHOP, Cyclophosphamide, Doxorubicin, Vincristine, Prednisone;FC, Fludarabine, Cyclophosphamide; Cl,Chlorambucil; Flu, Fludarabine; R, Rituximab; C, Cyclophosphamide; B, bendamustine; UNKN, unknown.

^a^Percentage of tumoral cells was quantified by flow cytometry labeling CD5+/CD19+ cells and light chain restriction.

^b^According to Rai and Binet’s classification: Early (Rai 0, Binet A), intermediate (Rai I/II, Binet B) and advanced (Rai III/IV, Binet C) stage disease.

^c^IGHV gene was sequenced following RT-PCR, and aligned to NCBI IgBlast. Mutated status was assigned when >2% deviation from germline IGHV sequence was present.

^d^ Cytogenetic alterations were assessed by FISH.

^e^Recurrent mutations were identified by exome-sequencing analysis within the ICGC project.

**Table S2. Flow cytometry antibodies**

| **Antibody** | Fluorophore | Clone | Vendor |
| --- | --- | --- | --- |
| Anti-mouse CD45 | AF700 | 30-F11 | Biolegend |
| Anti-mouse CD19 | PE-Cy7 or FITC | eBio1D3 | eBioscience |
| Anti-mouse CD5 | APC | 53-7.3 | Biolegend |
| Anti-mouse MHC-II I-A/I-E | AF700 or FITC | M5/114.15.2 | eBioscience |
| Anti-mouse CD25 | FITC or PerCP-Cy5.5 | PC61 | Biolegend |
| Anti-mouse CD40 | PE | 1C10 | eBioscience |
| Anti-mouse CD86 | PerCP-Cy5.5 | GL-1 | Biolegend |
| Anti-mouse CD69 | PE-Dazzle | H1.2F3 | Biolegend |
| Anti-mouse CD11b | PE-Cy7 | M1/70 | eBioscience |
| Anti-mouse F4/80 | APC or PE | BM8 | Biolegend |
| Anti-mouse CD54 | PE | YN1/1.7.4 | Biolegend |
| Anti-mouse Ly6G | FITC | 1A8 | Biolegend |
| Anti-mouse Ly6C | APC-Cy7 | HK1.4 | Biolegend |
| Anti-mouse PD-L1 | PerCP-eFluor710 | MIH5 | eBioscience |
| Anti-mouse CD3e | V450 | 500A2 | BD Biosciences |
| Anti-mouse CD90.2 | APC-Cy7 | 30-H12 | Biolegend |
| Anti-mouse CD4 | APC-Cy7 | RM4-5 | Biolegend |
| Anti-mouse CD8a | BV605 | 53-6.7 | Biolegend |
| Anti-mouse CD127 | PE-Cy7 | A7R34 | Biolegend |
| Anti-mouse CD44 | AF700 | IM7 | eBioscience |
| Anti-mouse CD28 | PE | 37.51 | Biolegend |
| Anti-mouse PD-1 | PE | RMPI-30 | Biolegend |
| Anti-mouse CD137 | APC | 17B5 | Biolegend |
| Anti-mouse CXCR3 | FITC | CXCR3-173 | eBioscience |
| Anti-mouse LAG3 | PE | eBioC9B7W | eBioscience |
| Anti-mouse TIGIT | PE-Dazzle | 1G9 | Biolegend |
| Anti-mouse CD160 | PerCP-Cy5.5 | 7H1 | Biolegend |
| Anti-mouse Granzyme B | V450 | NGZB | eBioscience |
| Anti-mouse Ki-67 | FITC | SolA15 | eBioscience |
| Anti-human CD14 | FITC | M5E2 | Biolegend |
| Anti-human PD-L1 | PE | MIH1 | eBioscience |
| Anti-human CD54 | PE-Vio770 | Not indicated | MiltenyiBiotec |
| Anti-human CD5 | FITC or APC | UCHT2 | Biolegend |
| Anti-human CD8a | PE-Dazzle or BV605 | RPA-T8 | Biolegend |
| Anti-human CD25 | BV605 | BC96 | Biolegend |
| Anti-human CD28 | PerCP-Cy5.5 | CD28.2 | Biolegend |
| Anti-human CD137 | PE | 4B4-1 | Biolegend |
| Anti-human Granzyme B | FITC | GB11 | Biolegend |
| Anti-human CD19 | PE | SJ25C1 | BD Biosciences |
| Anti-human CD3 | FITC | SK7 | BD Biosciences |
| Anti-human Ki-67 | PE-Cy7 | Ki-67 | Biolegend |

**Table S3. Gene set enrichment analysis of *MYD88*-mutated compared to *MYD88*-unmutated CLL cases.**

|  | ***MYD88* M (n=18) vs. *MYD88* UM (n=398)** | |  | ***MYD88* M IGHV M (n=18) vs.  *MYD88* UM IGHV M (n=249)** | |
| --- | --- | --- | --- | --- | --- |
|  | **^a^FDR** | **^b^NES** |  | **^a^FDR** | **^b^NES** |
| **TNFα signaling via NFκB** | **<0.0001** | **2,66** |  | **<0.0001** | **2.64** |
| **IL6 JAK STAT3 signaling** | **0.008** | **1,71** |  | **0.010** | **1.76** |
| **Inflammatory response** | **0.050** | **1,52** |  | **0.022** | **1.57** |
| **Apoptosis** | **0.039** | **1,51** |  | **0.012** | **1.69** |
| **IL2 STAT5 signaling** | **0.038** | **1,49** |  | **0.044** | **1.47** |
| **Hypoxia** | **0.131** | **1,30** |  | **0.021** | **1.59** |

^a^False discovery rate q-value (FDR)

^b^Normalized enrichment score (NES) ≥1.5 were considered to be significantly enriched in the mutated group.

M, mutated; UM, unmutated.

**SUPPLEMENTARY FIGURES**


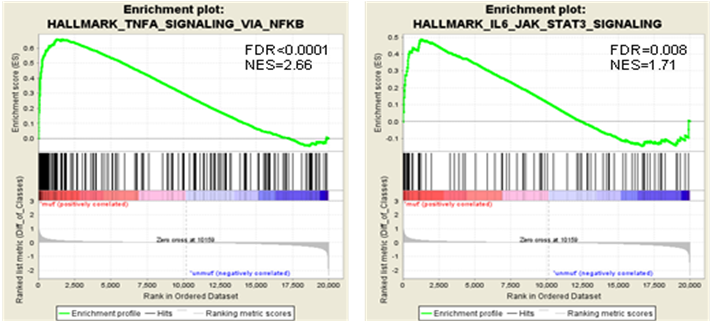


**Figure S1. Gene set enrichment analysis of *MYD88*-mutated and *MYD88*-unmutated CLL cases.** Enrichment plots for two gene sets that are significantly enriched in *MYD88*-mutated cases (n=18) compared to *MYD88*-unmutated CLL cases (n=398). Gene sets with false discovery rate (FDR) q-value <0.05 and a normalized enrichment score (NES) ≥1.5 were considered to be significantly enriched in the mutated group.

| **a.** |  |
| --- | --- |
|  | |
| **b.** | |

**Figure S2. TLR stimulation activates NF-κB and STAT3 signaling.** Western blot analysis of IκBα^pS32/36^ and STAT3^pY705^ phosphorylation levels in CLL cells after stimulation with TLR ligands for 3 h. **a)** Heatmap representing the mean rations of TLR-stimulated over untreated samples that were calculated from obtained densitometry values from 4 Western blots (3 *MYD88*-unmutated and 1 *MYD88*-mutated CLL samples). **b)** A representative *MYD88*-unmutated, IGHV-mutated CLL case (#31) is shown. α-tubulin levels were used as loading control.

**Figure S3.** Western blot results from Figure 2b including α-tubulin levels which was used as a loading control in each membrane.

| **a.** |
| --- |
| **b.** |
| **c.** |

**Figure S4. TLR stimulation increases cytokine secretion, NF-κB and STAT3 signaling of IGHV-unmutated CLL cells.** CLL cells were cultured with single TLR agonists or TLR agonist mix (Pam3CSK4, HKLM, FSL1 and ODN2006). **a)** Heatmap representing cytokine levels in CLL culture supernatants after 48 h of TLR stimulation (n=2) analyzed by flow cytometry Luminex® Bead Panel. The level of secretion of each cytokine is presented relative to untreated control. **b)** Western blot analysis of IκBα^pS32/36^ and STAT3^pY705^ phosphorylation and total levels of IκBα and STAT3 in IGHV-unmutated CLL cell extracts after 3 h of single or TLR agonist mix stimulation. α-tubulin was used as loading control. A representative IGHV-unmutated CLL case (#11) is shown. Ratios of phosphorylated and total protein levels were calculated and are provided as fold changes relative to the untreated control sample. **c)** Cytokine secretion after 48 h of TLR stimulation was assessed in cell culture supernatants by flow cytometry Luminex® Bead Panel (n=2). Data is presented as fold change relative to unstimulated control.


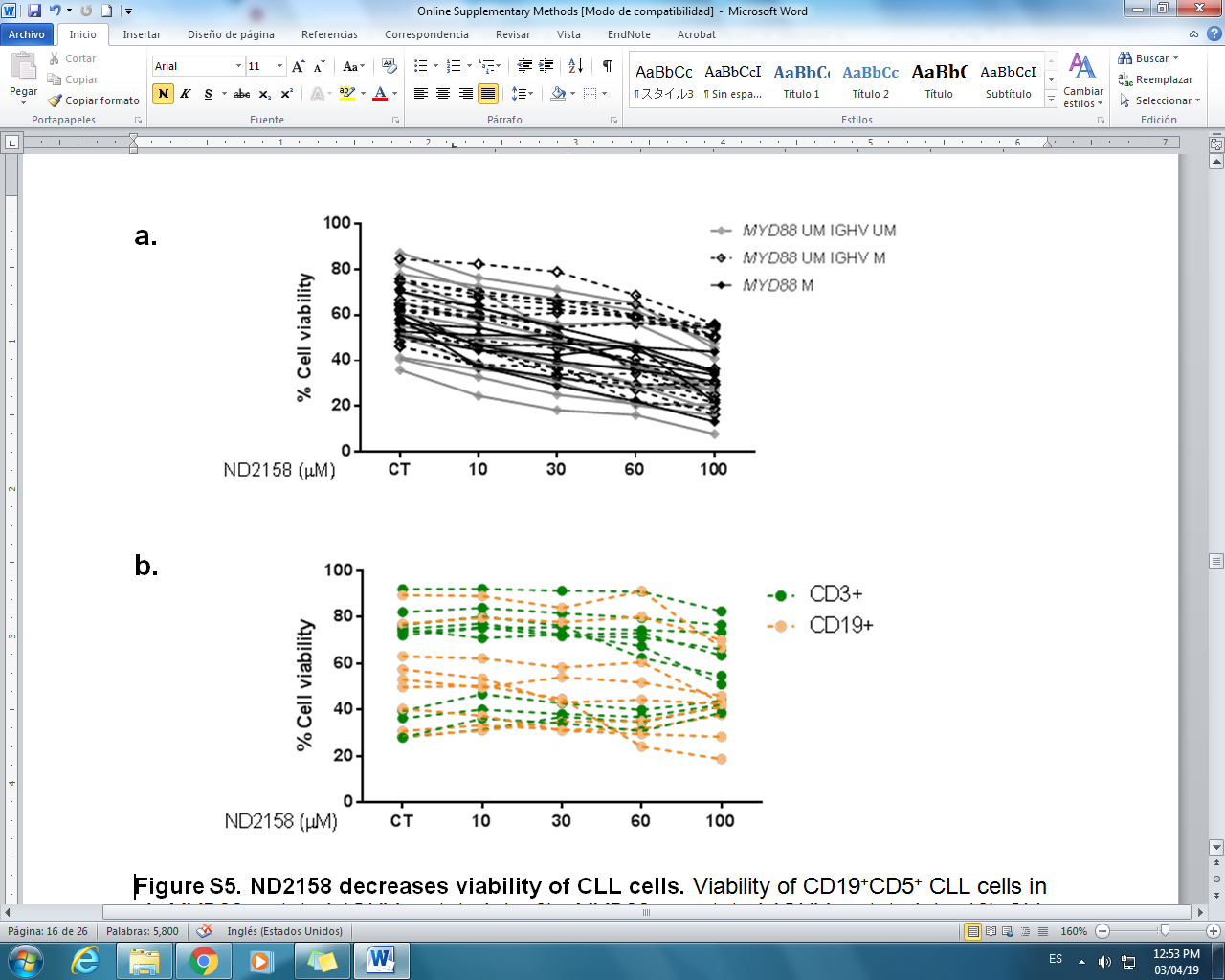


**Figure S5. ND2158 decreases viability of CLL cells.** Viability of CD19^+^CD5^+^ CLL cells in **a)** *MYD88-*mutated IGHV-mutated (n=6), *MYD88-*unmutated IGHV*-*mutated (n=16) CLL samples, and **b)** CD19^+^ B cells and CD3^+^ T cells from healthy donors (n=10) was analyzed by flow cytometry after 48 h of incubation with the indicated concentrations of ND2158. Percentage of viable cells was measured by staining with Annexin-V. M, mutated; UM, unmutated.


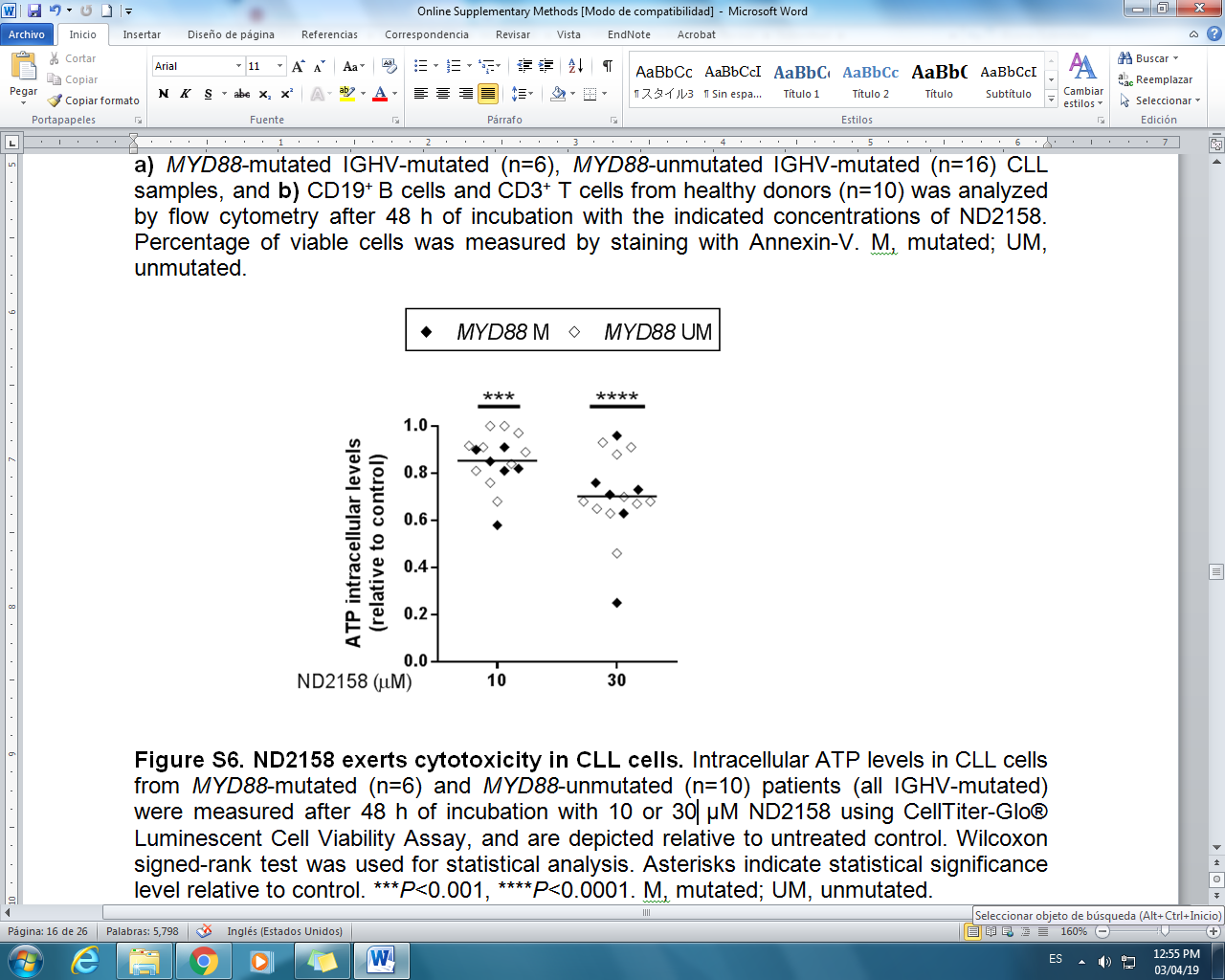


**Figure S6. ND2158 exerts cytotoxicity in CLL cells.** Intracellular ATP levels in CLL cells from *MYD88*-mutated (n=6) and *MYD88*-unmutated (n=10) patients (all IGHV-mutated) were measured after 48 h of incubation with 10 or 30 µM ND2158 using CellTiter-Glo® Luminescent Cell Viability Assay, and are depicted relative to untreated control. Wilcoxon signed-rank test was used for statistical analysis. Asterisks indicate statistical significance level relative to control. ****P*<0.001, *****P*<0.0001. M, mutated; UM, unmutated.


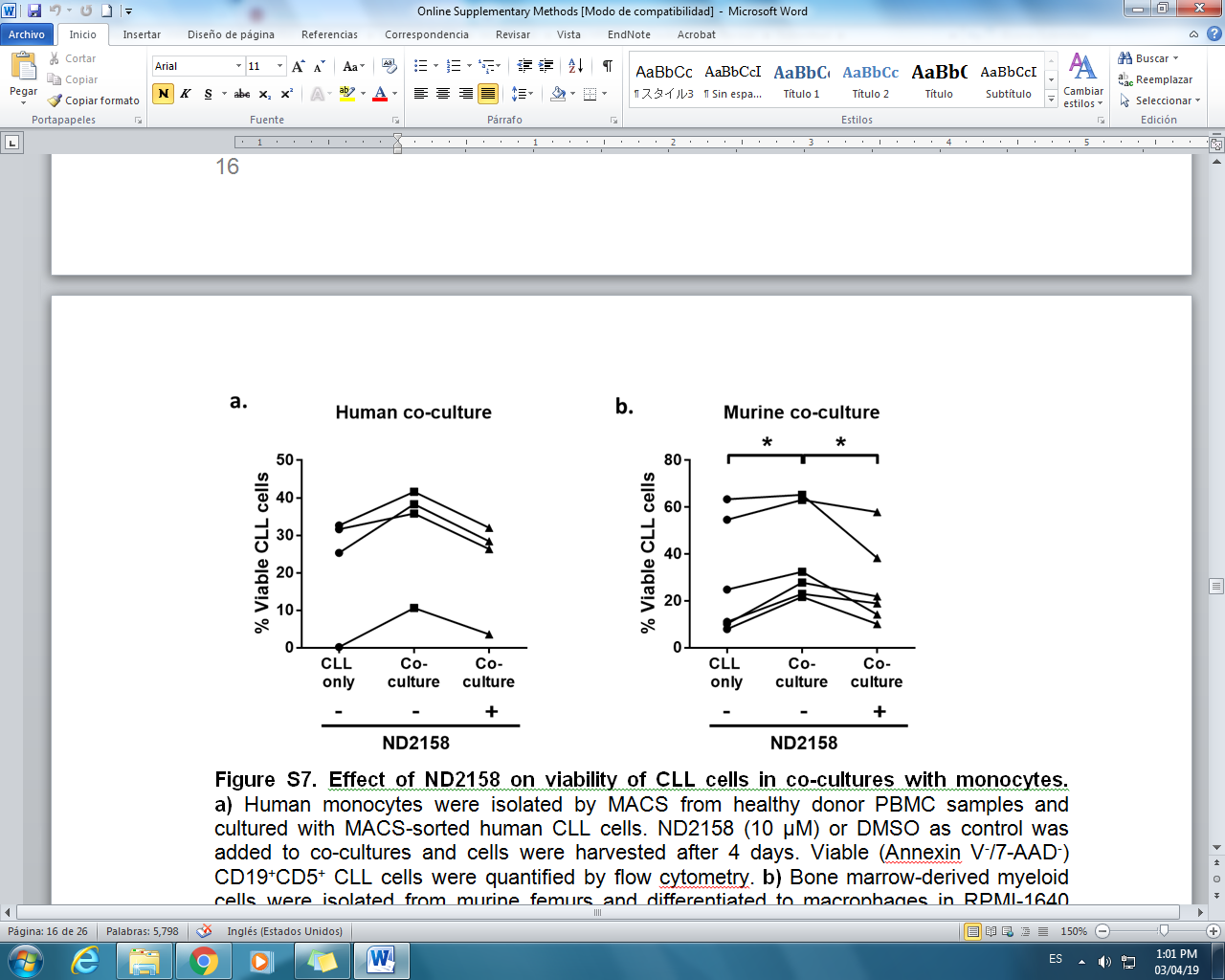


**Figure S7. Effect of ND2158 on viability of CLL cells in co-cultures with monocytes.** **a)** Human monocytes were isolated by MACS from healthy donor PBMC samples and cultured with MACS-sorted human CLL cells. ND2158 (10 µM) or DMSO as control was added to co-cultures and cells were harvested after 4 days. Viable (Annexin V^-^/7-AAD^-^) CD19^+^CD5^+^ CLL cells were quantified by flow cytometry. **b)** Bone marrow-derived myeloid cells were isolated from murine femurs and differentiated to macrophages in RPMI-1640 containing 30% L929 conditioned medium for 4 days. At day 4, malignant B cells isolated by MACS from the spleen of mice with TCL1 leukemia were added to bone marrow-derived macrophages and treated with 10 µM ND2158 or DMSO. Viable (Annexin V^-^/7-AAD^-^) CD19^+^CD5^+^ CLL cells were quantified by flow cytometry after 48 h of co-culture. Wilcoxon matched pairs signed-rank test was used for statistical analysis. **P*<0.05.

**Figure S8. Anti-proliferative effect of ND2158 on TLR-stimulated CLL cells *in vitro*.** CLL cells were exposed to TLR agonists and IL15 for 30 min before adding 10 µM ND2158. Proliferation of cells was analyzed by CFSE dilution assay after 6 days of incubation. Histograms of a representative *MYD88*-unmutated, IGHV-mutated CLL case (#51) are shown.

| **a.** | **b.** |
| --- | --- |
|  |  |

**Figure S9.** Percentage of proliferating CD19^+^CD5^+^ CLL cells after TLR stimulation and 10 µM ND2158 treatment for 6 days measured by **a)** EdU incorporation or **b)** Ki-67^+^ staining (n=6). Wilcoxon matched pairs signed rank test was used for statistical analysis. **P*<0.05. M, mutated; UM, unmutated.

**Figure S10. NF-κB basal levels in *MYD88*-mutated and *MYD88*-unmutated CLL cases.** Basal levels of p65 and p52 NF-kB subunits in nuclear extracts of *MYD88*-mutated (n=4) and *MYD88*-unmutated cases (n=4) were determined by NF-κB DNA-binding assay to plate-bound NF-kB consensus sequence oligos after 3 h of incubation. Obtained values for NF-kB binding are presented relative to *MYD88*-unmutated mean values. Results are expressed as mean ± SD. Mann-Whitney test was used for statistical analysis. ns, not significant; *P*≥0.05, **P*<0.05. M, mutated; UM, unmutated.

**Figure S11.** Western blot results from Figure 4b including α-tubulin levels which was used as a loading control in each membrane.

**Figure S12. Inhibition of cytokine secretion by ND2158 in TLR-stimulated CLL cells *in vitro*.** Cells were exposed to TLR agonists for 30 min before adding 10 µM ND2158. Cytokine levels of CCL2, CCL3, CCL4, TNFα, IL1β and IL6 in supernatants from *MYD88*-mutated (n=2) and *MYD88*-unmutated (n=3) CLL cases after 48 h of culture were analyzed. Values are depicted relative to untreated control. Wilcoxon signed-rank test was used for statistical analysis. *****P*<0.0001.

| a. |
| --- |
| b. |
| c. |

**Figure S13. Impact of ND2158 on IGHV-unmutated CLL cells. a) Left panel:** Viability of IGHV-unmutated CLL cells (n=15) was analyzed by flow cytometry after 48 h of incubation with the indicated concentrations of ND2158. Percentage of viable cells was measured by staining with Annexin-V and normalized to untreated control. **Right panel:** Intracellular ATP levels after 48 h of 10 µM and 30 µM ND2158 treatment relative to untreated control (n=7). **b)** **Left panel**: Viability of ND2158-treated CLL cells was analyzed after TLR stimulation for 2 (n=6) and 6 days (n=4). **Right panel**: Percentage of proliferating CD19^+^ CLL cells after TLR stimulation and ND2158 treatment for 6 days measured by CFSE dilution (n=3). **c)** Cytokine levels in supernatants from 4 samples exposed to TLR agonist mix prior treatment with ND2158 for 48 h was analyzed by a flow cytometry using Luminex® Bead Panel. Values are presented relative to untreated control. **d)** Migration of TLR-stimulated CLL cells treated with ND2158 (n=3) towards CXCL12 was analyzed by transwell assays after 3 h of incubation. Values are presented as the ratio of migrating cells and total viable cells, relative to the non-stimulated, untreated control.

**a.**

**b.**

**c.**

**Figure S14. ND2158 blocks proliferation of leukemic B cells from TCL1 mice. a)** Intracellular ATP levels of TCL1 splenocytes after 48 h of incubation with TLR agonist mix and 10 µM and 30 µM ND2158 relative to untreated controls (n=6). Results are expressed as mean ± SEM. Wilcoxon signed-rank test was used for statistical analysis. **b)** Quantification of flow cytometry data of TCL1 splenocytes showing percentages of viable CD45^+^CD19^+^CD5^+^ cells with a decrease in CFSE staining, indicative of new cell generations, after 3 days of incubation with TLR ligands or the TLR agonist mix, and treatment with 10 µM ND2158 or without treatment (n=9). **c)** CFSE histograms of one representative sample of TCL1 splenocytes treated as described above; non-stimulated cells were used as control. **P*<0.05, ***P*<0.01. FMO, fluorescence-minus-one.

**
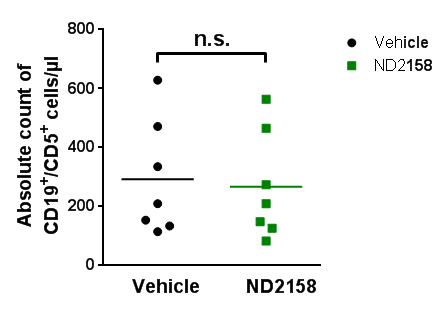
**

**Figure S15. Assignment of mice to treatment arms based on number of leukemic cells in blood.** Absolute counts of CD19^+^CD5^+^ CLL cells in the peripheral blood of TCL1 AT mice assigned to vehicle (n=7) or ND2158 (n=7) treatment groups 13 days after tumor transplantation and 1 day before treatment start. Mann-Whitney test was used for statistical analysis. n.s., not significant; *P*≥0.05.

**Figure S16. Absolute tumor cell count in the spleen of TCL1 adoptive transfer mice after treatment with ND2158.** After 23 days of treatment, absolute count of tumor cells (CD19^+^CD5^+^) in the spleen of vehicle- (n=7) and ND2158-treated (n=7) mice was analyzed.


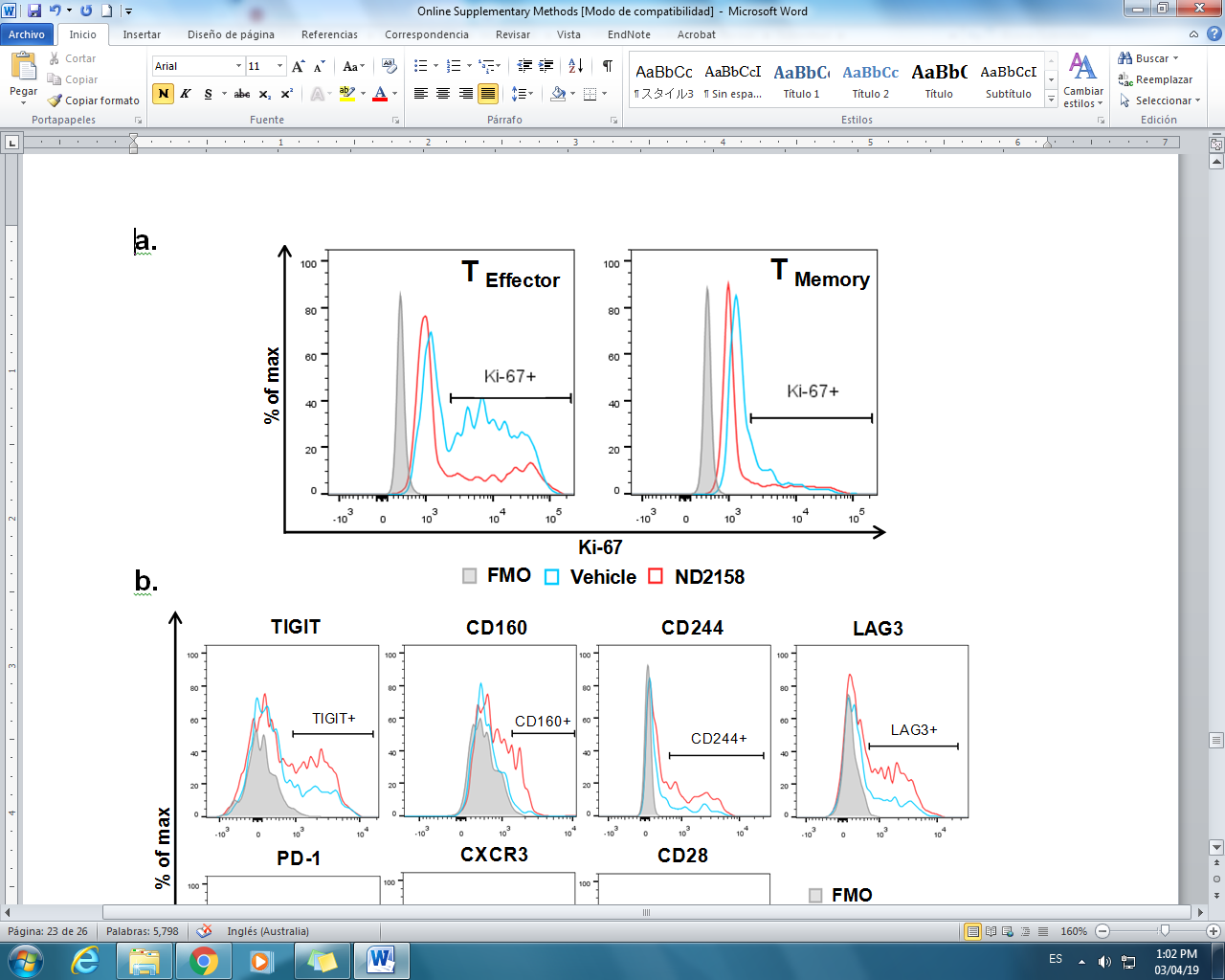
**a.**

**b.**

**c.**

**Figure S17. Effects of ND2158 treatment on CD8^+^ T cells in the TCL1 AT mouse model. a)** Representative flow cytometry histograms of Ki-67^+^ levels in CD8^+^ effector and CD8^+^ memory T cells from the spleen of vehicle- (n=7) or ND2158-treated (n=7) mice. **b)** Representative flow cytometry histograms of TIGIT, CD160, CD244, LAG3, PD-1, CXCR3 and CD28 –expression in splenic CD8^+^ effector T cells from ND2158 or vehicle-treated mice. **c)** Percentage of PD-1-expressing CD8^+^ effector T cells in the spleen of vehicle- (n=7) or ND2158-treated (n=7) mice as determined by flow cytometry. Mann-Whitney test was used for statistical analysis. n.s, not significant; *P*≥0.05, **P*<0.05, ***P*<0.01. MFI, median fluorescence intensity; FMO, fluorescence-minus-one.

**a.**

**b.**

**c.**

**Figure S18. ND2158 impairs CD8^+^ T cell proliferation, activation and T cell receptor signaling *in vitro*. a)** Splenocytes from WT C57BL/6 mice (n=8) were stained with CFSE and stimulated with an anti-CD3 antibody 30 min before adding 10 µM ND2158. **Left panel**: Percentage of proliferating cells as measured by CFSE dilution was analyzed in viable single CD8^+^ T cells after 2 days. Wilcoxon matched pairs signed-rank test was used for statistical analysis. **Right panel**: a representative histogram is shown. **b)** Splenocytes from WT C57BL/6 (n=4) mice were stimulated with an anti-CD3 antibody 30 min before adding 10 µM ND2158. Protein expression of CD25, CD28, CD137 and granzyme B was analyzed after 24 h in viable single CD8^+^ T cells by flow cytometry. Granzyme B expression was analyzed after restimulation with PMA and ionomycin and addition of a protein transport inhibitor 4 h before harvesting the cells. Quantifications are shown in the upper row; corresponding representative histograms are shown in the bottom row. Data are shown as percentage for bimodal populations or as MFI of CD8^+^ T cells for unimodal populations. **c)** Representative histogram of GFP fluorescence from splenocytes of *Nr4a1^GFP^* transgenic mice (n=4). Cells were stimulated with an anti-CD3 antibody 30 min before adding 10 µM ND2158. GFP expression was analyzed by flow cytometry in viable single CD8^+^ T cells after 3 h. ***P*<0.01. MFI, median fluorescence intensity; FMO, fluorescence-minus-one.

**Figure S19. ND2158 impairs activation of *Myd88*-deficient CD8^+^ T cells *in vitro.*** Splenocytes from WT C57BL/6 (n=4) and *Myd88^–/–^* mice (n=4) were stimulated with anti-CD3 antibody 30 min before 10 µM ND2158 was added. Protein expression of CD25, CD137, CD28 and granzyme B was analyzed after 24 h in viable, single CD8^+^ T cells from WT (**Left panel**) or *Myd88*^-/-^ mice (**Right panel**) by flow cytometry. Granzyme B expression was analyzed after restimulation with PMA and ionomycin and addition of a protein transport inhibitor 4 h before harvesting the cells. Data are shown as percentage of CD25^+^, CD137^+^ or granzyme B^+^ CD8^+^ T cells for bimodal populations, or as MFI of CD28 of CD8^+^ T cells as unimodal population. MFI, median fluorescence intensity; WT, wild-type.


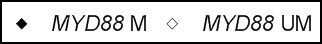


**a.**

**b.**

**Figure S20. Combinatory effects of ND2158 and venetoclax or ibrutinib.** CLL cells were stimulated *in vitro* with TLR agonist mix for 30 min, before 10 µM ND2158, 1 nM venetoclax or 0.25 µM Ibrutinib were added to the cultures as indicated. Percentage of viable cells was measured as CD19^+^Annexin-V^-^ cells by flow cytometry and normalized to untreated control samples. **a)** Single treatment with ND2158 or venetoclax or their combination was measured after 48 h of incubation of CLL cells from *MYD88*-mutated (n=3) and *MYD88*-unmutated (n=4) patients (IGHV-mutated). **b)** Single treatment with ND2158 or ibrutinib or their combination was measured after 6 days of incubation of CLL cells from *MYD88*-mutated (n=4) and *MYD88*-unmutated (n=4) patients (IGHV-mutated). Wilcoxon signed-rank test was used for statistical analysis. **P*<0.05, ***P*<0.01.
